# HLA Variants and Inhibitor Development in Hemophilia A: A Retrospective Case-Controlled Study Using the ATHNdataset

**DOI:** 10.3389/fmed.2021.663396

**Published:** 2021-05-07

**Authors:** Joseph R. McGill, Vijaya L. Simhadri, Zuben E. Sauna

**Affiliations:** Hemostasis Branch, Division of Plasma Protein Therapeutics, Center for Biologics Evaluation and Research, Food and Drug Administration, Silver Spring, MD, United States

**Keywords:** inhibitors, factor VIII, HLA-type, statistics, hemophilia, ATHN, MLOF

## Abstract

In hemophilia A (HA) patients, *F8* gene-defects as genetic risk-factors for developing inhibitors to Factor VIII have been extensively studied. Here we provide estimates of inhibitor-risk associated with the patient's Human Leukocyte Antigen (HLA). We used next generation sequencing for high-resolution HLA Class II typing of 997 HA patients. Using inhibitor prevalence reports from the My Life Our Future (MLOF) research repository, we calculated Odds Ratios (OR) for inhibitor development in a multivariate model considering HLA-DRB1/3/4/5, HLA-DPB1, HLA-DQB1, race, *F8* pathogenic variant type, and age. Participants with 1 HLA variant (DPB1*02:02) had developed inhibitors at a higher rate while participants with 2 HLA variants (DRB1*04:07; DRB1*11:04) had developed inhibitors at a lower rate. Additionally, patients with missense variants had developed inhibitors at a lower rate and participants with large structural changes (>50 bp) had developed inhibitors at a higher rate (both compared to Intron 22 inversion). Using a cohort of participants with a distribution of HLA-DRB1 alleles comparable to that in the North American population we show that the HLA repertoire of a HA patient can be a risk-factor for inhibitor development.

## 1. Introduction

An unmet need in the management of hemophilia-A (HA) is the lack of clinically validated markers associated with the development of inhibitors, i.e., neutralizing antibodies to Factor VIII (FVIII). Approximately 20% of HA patients and 30% of severe HA patients develop inhibitors which represent an impediment to the effective management of HA (1, 2). The availability of markers for immunogenicity would prove useful for more efficient clinical care and personalization of the treatment of HA patients. Inhibitors are also the key safety concern during drug development and licensure; the absence of non-clinical markers means that immunogenicity assessments can only be made as part of phase three studies; the most expensive phase of drug development.

There is broad recognition that genetic factors play a role in determining which patients develop inhibitors and which do not (3,8). However, identifying the genetic markers of immunogenicity is challenging. For instance, there is evidence that CD4+ T-cell response is essential for eliciting inhibitors (9). It is a reasonable assumption that presentation of the peptides by the MHC-class-II (MHC-II) molecules [human leukocyte antigens (HLA), in humans] is a necessary step in the immune cascade that results in inhibitory antibodies. There have been several studies to identify HLA variants potentially associated with inhibitors, however no consistent correlates were found between studies (10,14). These studies were all performed with small sample sizes ranging from 57 to 176 participants. These sample sizes are inadequate for making meaningful, statistically powered, assessments, considering that the MHC region, containing 164 HLA genes is the most polymorphic in the human genome with over 11,000 variants reported (15).

The HLA is not the only genetic risk-factor implicated with inhibitors to FVIII. HA is caused by variants in the *F8* gene that range from missense variants to large deletions (5). An earlier meta-analysis (of 30 independent studies and 5,383 participants) showed that larger gene disruptions (e.g., deletion of multiple exons) were associated with a higher OR of developing inhibitors (5). Although the meta-analysis did provide a considerably larger total cohort than any individual study, the approach suffers from some disadvantages. Meta-analyses often fail to control for the fixed effects attributable to different testing centers and the number of participants in each study. One possible outcome of this is Simpson's Paradox in which trends identified in the individual studies cannot be found in the pooled data (16). In addition, different studies often target participants with specific variants (e.g., Intron 22 Inversion) or specific populations. The differing baseline risks for these groups will further lead to heterogeneous population groups which require careful analysis to avoid biases. Furthermore, meta-analyses relies solely on previously published studies and will suffer from publication bias and possibly exaggerate results by not considering the unavailable, unpublished data (17). Consequently, meta-analyses are often considered more suitable for hypothesis generation than for hypothesis testing (18).

A large cohort of HA patients who are genotyped using consistent methods and for whom clinical information is available is a clear unmet need. The My Life, Our Future (MLOF) project [a collaboration between the American Thrombosis and Hemostasis Network (ATHN), National Hemophilia Foundation (NHF), Bloodworks Northwest and Bioverativ] provided free hemophilia genotype analysis for participants in the United States. As the MLOF collaboration did not HLA type the participants, we have HLA typed 1,000 participants for whom *F8* genotype and clinical and demographic information was available. This data set is at least four-times larger than those used in published studies and is adequate to assess the association between HLA type and inhibitors. We found the HLA variant DPB1*02:02 is associated with higher odds of eliciting inhibitors to FVIII. The HLA variants DRB1*04:07 and DRB1*11:04 are associated with lower odds of developing inhibitors. With respect to pathogenic *F8* variants, our results are consistent with the previous conclusion from a meta-analysis. Compared to participants with the intron-22-inversion, those with missense variants have significantly lower odds of inhibitor formation. Conversely, participants with large structural changes (>50 bp) show significantly higher odds of developing inhibitors. We also show that Hispanic participants had a higher prevalence of inhibitors.

## 2. Materials and Methods

### 2.1. Study Design

This is a retrospective case-controlled study. Data from the ATHNdataset was merged with HLA-typing data obtained by us for 997 participants. Phenotypic and genetic features were compared to the prevalence of inhibitor development in these participants. Statistical analysis was performed as a series of univariate logistic regression model that would determine inclusion of a variable in a multivariate logistic regression model.

### 2.2. Data Sources

The MLOF program is the result of a collaboration between the ATHN, NHF, and Bloodworks Northwest, with support of Bioverativ through June 2018. Participants and/or their parents gave written informed consent for inclusion of their samples and data in the MLOF Research Repository. Phenotypic data on MLOF Research Repository participants was abstracted from the ATHNdataset collected from participating hemophilia treatment centers around the United States, including demographic, phenotypic, and genomic data. Participants self-reported their race and ethnicity (19).

The background distribution of HLA-DRB1 Alleles was obtained using a population weighted according to US Census estimates of population demographics from July, 2018 (20).

### 2.3. Determinations of Hemophilia Severity and Inhibitor Development

Hemophilia Severity was identified based on reported FVIII baseline activity (percent of normal) in the ATHNdataset based on the following criteria: FVIII activity 1%, Severe; FVIII activity 5% but >1%, Moderate and FVIII activity >5%, Mild. Factor VIII activity was tracked using the lowest value that can be historically tracked. The assays were run in independent clinical laboratories and were primarily one-stage assays.

### 2.4. HLA Testing for Class II Loci Using Next Generation Sequencing

We used Next Generation Sequencing (NGS) as it offers robust HLA testing by increasing typing resolution visvis Sanger sequencing methods. DNA barcoding and single molecule sequencing were used to allow for better efficiency and economies of scale (21). LabCorp designed a test to sequence participant samples concurrently for Class II HLA loci DRB1/3/4/5, DQB1, and DPB1 by NGS. The validation was conducted using an open-platform and Illumina MiSeq analyzers. The gene coverage for the targeted NGS assay represents the Antigen Recognition Domain which is encoded in exon 2 for the MHC Class II (22).

### 2.5. Determining the Size of the Cohort Used for HLA Typing

No hard-and-fast rule exists for the selection of sample sizes for multivariate logistic regression; a general rule of thumb is that 1030 samples are adequate to test the impact of a particular factor with sufficient statistical power (23). However, due to the heterogeneous distribution of alleles, some alleles, would easily be found with a frequency of 30, other alleles [e.g., HLA-DRB1*04:38 (0.0005%)] (20), would never be found in sufficient numbers for adequate statistical analysis regardless of the cohort size.

A list of 38 MHC-DRB1 alleles (20) was chosen to represent 99% of the North American Population and create a reasonable pool of alleles that would be found in the MLOF Research Repository cohort. A simulation was run generating 100 cohorts each of various sizes by randomly assigning alleles based on their frequencies in the North American population. We counted both the number of alleles that would be found in at least 30 participants as well as the population coverage of those alleles. Cohorts of sizes from 100 to 1,500 were generated and the population coverage of alleles which occurred in 30 or more individuals was recorded.

### 2.6. Filtering the Data

**Figure 1:**
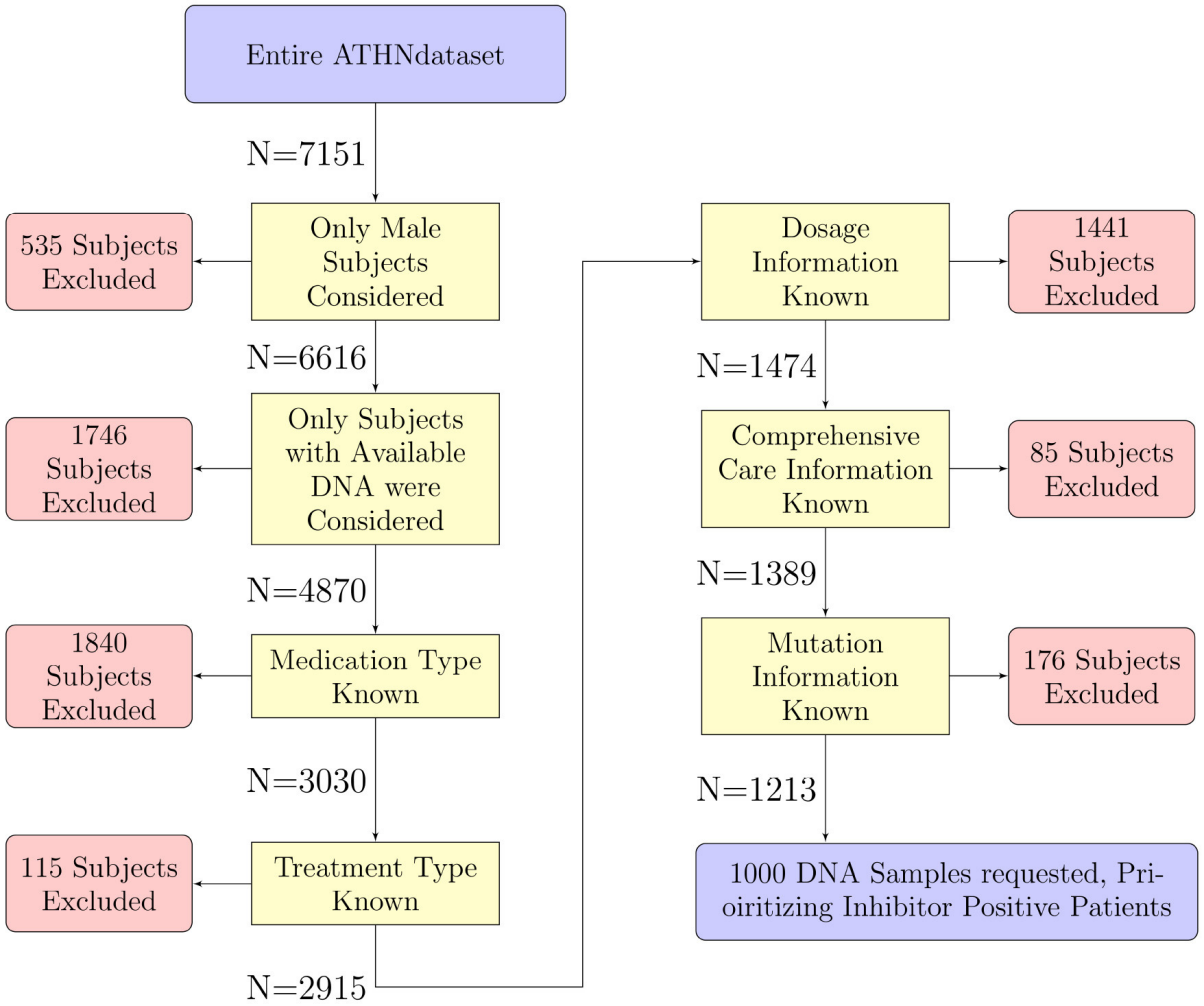
Selection of participants for HLA typing. The 7,151 participants in the entire ATHNdataset were filtered to select participants suitable for further analysis. We have filtered out participants for sex, availability of DNA for HLA-typing, and lack of clinical or genetic information. There were 1,213 participants which met all our criteria. One thousand of these participants' DNA were sent for HLA typing.

As it was not feasible to HLA type the entire cohort of 7,151 donors, a subset of 1,000 participants were chosen for HLA typing. Donors were filtered out for the following reasons (Figure 1):

Sex: Only participants who were listed as male were included in this analysis. The gene coding for the FVIII protein is located on the X chromosome, thus inclusion of female participants would have introduced confounding factors such as genetic carriers as well as introduce another confounding variable into the analysis.

Available DNA: Only participants with DNA available for HLA typing were considered.

Medication type, treatment type, dosage information, comprehensive care information, and pathogenic variant: We included only individuals for who clinical and genetic information was available.

After filtering the list of participants for inclusion in our HLA typed cohort, 1,213 participants remained: 958 without inhibitors and 255 with inhibitors. In order to enrich the population of inhibitor positive participants to match the proportion of inhibitor positive participants in the HA population (30%) we used stratified sampling (24). This method samples from different strata with different frequencies. We split the remaining participants into two strata based on inhibitor status. We carried out HLA typing on 1,000 participants, prioritizing selection of the inhibitor positive participants. This method was used to help bring the proportion of inhibitor positive samples to the desired 30%. As this is a case-controlled study, and our analysis relies on odds-ratios rather than relative risk or prevalence, we decided on this method to increase the frequency of observing inhibitor development. With this increase in frequency of inhibitor development observed, we stand a greater chance of observing events in conjunction with rare alleles.

### 2.7. Missing Data

Of the 1,000 participants sent for HLA typing: three participants had missing HLA type data (0.3%), 6 (0.6%) had missing data for race, and three participants had missing data for FVIII variant type. In total <5% of data was missing.

### 2.8. Statistical Analysis

All HLA-DRB1/3/4/5, HLA-DPB1, HLA-DQB1, race, ethnicity, disease severity, and pathogenic variant type were analyzed using univariate logistic regression models. For each of the HLA variants, a participant was considered as having that variant if at least one of the alleles matched. For race and variant type, ORs were calculated compared to reference levels; White for race and intron-22 inversion for variant type. Variables with some degree (*p* < 0.25) of significant correlation to the odds of inhibitor development were included in a multivariate model using Hosmer and Lemeshow's guidance on purposeful variable selection (23).

Log likelihood analysis was used to determine the appropriateness of adding age into the explanatory model as both a linear variable and a third-degree polynomial. The log likelihood looks at the difference between a model including age as a linear predictor as well as age-squared and age-cubed and a model with age only as a linear predictor. This difference is compared to a ^2^ distribution with degrees of freedom equal to the number of additional variables. While the addition of additional variables will necessarily increase the likelihood of a model, the ^2^ test helps to only include variables which are adding significant increases to the goodness-of-fit of the model.

*P*-values from the multivariate model were adjusted using the Benjamini-Hochberg method (25) for controlling the rate of false discoveries. The adjusted *p*-value reported represents the strictest false discovery rate for which a particular hypothesis will be rejected using the Benjamini-Hochberg method as used in the R (26) function p.adjust (27). An adjusted *p*-value presented here can be directly compared to using a desired false discovery rate of 0.05 as an acceptance criterion for a hypothesis test and will yield equivalent results to calculating individual *p*-value thresholds for each of multiple hypotheses.

This procedure was also repeated for a subset of the study cohort which had severe hemophilia omitting the variable coding for disease severity. This subset of only severe HA participants was used for our primary analysis.

All statistical analysis was done using the R programming language (26) and all graphics were produced using ggplot2 (28). All tables were produced using kable (29) and LaTeX (30).

### 2.9. Predicted Binding Affinity of Foreign Sequences at the Location of Missense Mutation in the *F8* Gene of Study Participants

For all study participants with a missense mutation, a list of FVIII sequences that would be foreign for each participant was generated. This is the wild type sequence (found in the infused FVIII drug) at that location and is foreign to a participant with the missense mutation. We then used netMHCIIpan version 3.2 (31) to estimate the binding affinities of all foreign peptides in the region of the missense mutations to the HLA-DRB1 alleles identified in that participant. The binding affinities were reported as percentile ranks. The minimum percentile rank (highest affinity) for each participant was used in determining if binding affinities are significantly higher for those individuals with inhibitors. We used the Shapiro-Wilk test and the results were compared using a one-sided MannWhitney *U*-test testing the hypothesis that the percentile rank scores of participants who had developed an inhibitor would be lower than participants who had not developed inhibitors.

## 3. Results

### 3.1. Selecting a Representative Cohort Size

Per our simulation, a sample size of 1,000 participants provides adequate coverage of HLA variants in all 100 runs. In these runs, 97.3% of the allele population of North America was expected to be found in sufficient numbers for analysis.

### 3.2. Participant Characteristics

We have presented a detailed breakdown of participant characteristics for the entire ATHNdataset (*N* = 7,151), The subset of HLA-typed participants with Severe HA (*N* = 612), and the HLA-typed subset (*N* = 997) (Tables 1^3^). We have additionally compared the characteristics of the HLA-type group as a whole with the entire ATHNdataset to ensure that there was no bias in the cohort selection other than the enrichment of inhibitor positive cases (Supplementary Table 1).

**Table 1:** Participant characteristics, the ATHNdataset.

	**Inhibitor**		**Inhibitor**	
	**negative**		**positive ever**	
	**Number**	**%**	**Number**	**%**
**Number of participants**	6,028		1,123	
**Disease severity**				
Mild	2,229	36.98	73	6.50
Moderate	922	15.30	86	7.66
Severe	2,877	47.73	964	85.84
**Primary treatment type**				
Episodic	1,665	27.62	175	15.58
Immune tolerance induction	6	0.10	36	3.21
Prophylaxis	2,065	34.26	555	49.42
Unknown	2,292	38.02	357	31.79
**Race/Ethnicity**				
American Indian or Alaska	71	1.18	6	0.53
Native				
Asian	226	3.75	58	5.16
Black or African American	520	8.63	189	16.83
non-hispanic				
Hispanic	1,028	17.05	208	18.52
Mixed race	58	0.96	12	1.07
Native Hawaiian or other	21	0.35	3	0.27
Pacific Islander				
White non-hispanic	4,032	66.89	636	56.63
None reported	72	1.19	11	0.98
**Age (years)**				
<5	366	6.07	95	8.46
514	1,462	24.25	340	30.28
1524	1,427	23.67	279	24.84
2539	1,403	23.27	236	21.02
4064	1,042	17.29	136	12.11
65	328	5.44	37	3.29
**Variant type**				
5 Upstream	11	0.18	0	0.00
Frameshift	491	8.15	112	9.97
Intron 1 inversion	48	0.80	17	1.51
Intron 22 inversion	1,089	18.07	440	39.18
Large structural change^*a*^	148	2.46	85	7.57
Small structural change^*b*^	33	0.55	2	0.18
Missense	2,595	43.05	162	14.43
Nonsense	301	4.99	108	9.62
Splice site change	151	2.50	33	2.94
Synonymous	172	2.85	2	0.18
Untranslated region	2	0.03	0	0.00
None reported	987	16.37	162	14.43

a *denotes 50 bp*.

b *denotes <50 bp*.

**Table 2:** Participant characteristics, HLA typed participants with severe hemophilia A.

	**Inhibitor**		**Inhibitor**	
	**negative**		**positive ever**	
	**Number**	**%**	**Number**	**%**
**Number of participants**	395		217	
**Primary treatment type**				
Episodic	43	10.89	35	16.13
Immune tolerance induction	0	0.00	5	2.30
Prophylaxis	352	89.11	177	81.57
**Race/Ethnicity**				
American Indian or Native	6	1.52	0	0.00
Alaskan				
Asian	22	5.57	12	5.53
Black or African American	48	12.15	35	16.13
non-Hispanic				
Hispanic	40	10.13	39	17.97
Mixed race	5	1.27	2	0.92
Native Hawaiian or other	2	0.51	1	0.46
Pacific Islander				
White non-hispanic	270	68.35	127	58.52
None reported	2	0.51	1	0.46
**Age (years)**				
<5	1	0.25	16	7.37
514	98	24.81	77	35.48
1524	115	29.11	50	23.04
2539	119	30.13	46	21.20
4064	54	13.67	28	12.90
65	8	2.03	0	0.00
**Variant type**				
5 Upstream	0	0.00	0	0.00
Frameshift	71	17.97	30	13.82
Intron 1 inversion	8	2.03	7	3.23
Intron 22 inversion	154	38.98	104	47.92
Large structural change^*a*^	12	3.03	21	9.68
Small structural change^*b*^	4	1.01	1	0.46
Missense	93	23.54	15	6.91
Nonsense	42	10.63	32	14.75
Splice site change	11	2.78	7	3.23
Synonymous	0	0.00	0	0.00

a *denotes 50 bp*.

b *denotes <50 bp*.

**Table 3:** Participant characteristics, HLA typed participants.

	**Inhibitor**		**Inhibitor**	
	**negative**		**positive ever**	
	**Number**	**%**	**Number**	**%**
**Number of participants**	745		252	
**Disease severity**				
Mild	231	31.01	18	7.14
Moderate	119	15.97	17	6.75
Severe	395	53.02	217	86.11
**Primary treatment type**				
Episodic	333	44.70	60	23.81
Immune tolerance induction	0	0.00	7	2.78
Prophylaxis	412	55.30	185	73.41
**Race/Ethnicity**				
American Indian or Native	7	0.94	0	0.00
Alaskan				
Asian	29	3.89	14	5.56
Black or African American	66	8.86	40	15.87
non-hispanic				
Hispanic	102	13.69	39	15.48
Mixed race	7	0.94	2	0.79
Native Hawaiian or other	2	0.27	1	0.40
Pacific Islander				
White non-hispanic	527	70.74	155	61.51
None reported	5	0.67	1	0.40
**Age (years)**				
<5	2	0.27	17	6.75
514	181	24.30	82	32.54
1524	212	28.46	61	24.21
2539	184	24.70	55	21.83
4064	122	16.38	33	13.10
65	44	5.91	4	1.59
**Variant type**				
5 Upstream	2	0.27	0	0.00
Frameshift	75	10.07	31	12.30
Intron 1 inversion	9	1.21	7	2.78
Intron 22 inversion	160	21.48	107	42.46
Large structural change^*a*^	13	1.74	22	8.73
Small structural change^*b*^	4	0.54	1	0.40
Missense	394	52.89	45	17.86
Nonsense	44	5.91	32	12.70
Splice site change	16	2.15	7	2.78
Synonymous	25	3.36	0	0.00
None reported	3	0.40	0	0.00

a *denotes 50 bp*.

b *denotes <50 bp*.

The entire cohort studied was 7,151 Hemophilia A participants. Of these participants, 1,123 (15.7%) had developed inhibitory antibodies. In the subset of 612 severe HA participants, 217 (35.5%) had developed inhibitors. In the subset of 997 HLA-typed participants, 252 (25.28%) had developed inhibitory antibodies.

### 3.3. HLA Typing of Participants

**Figure 2:**
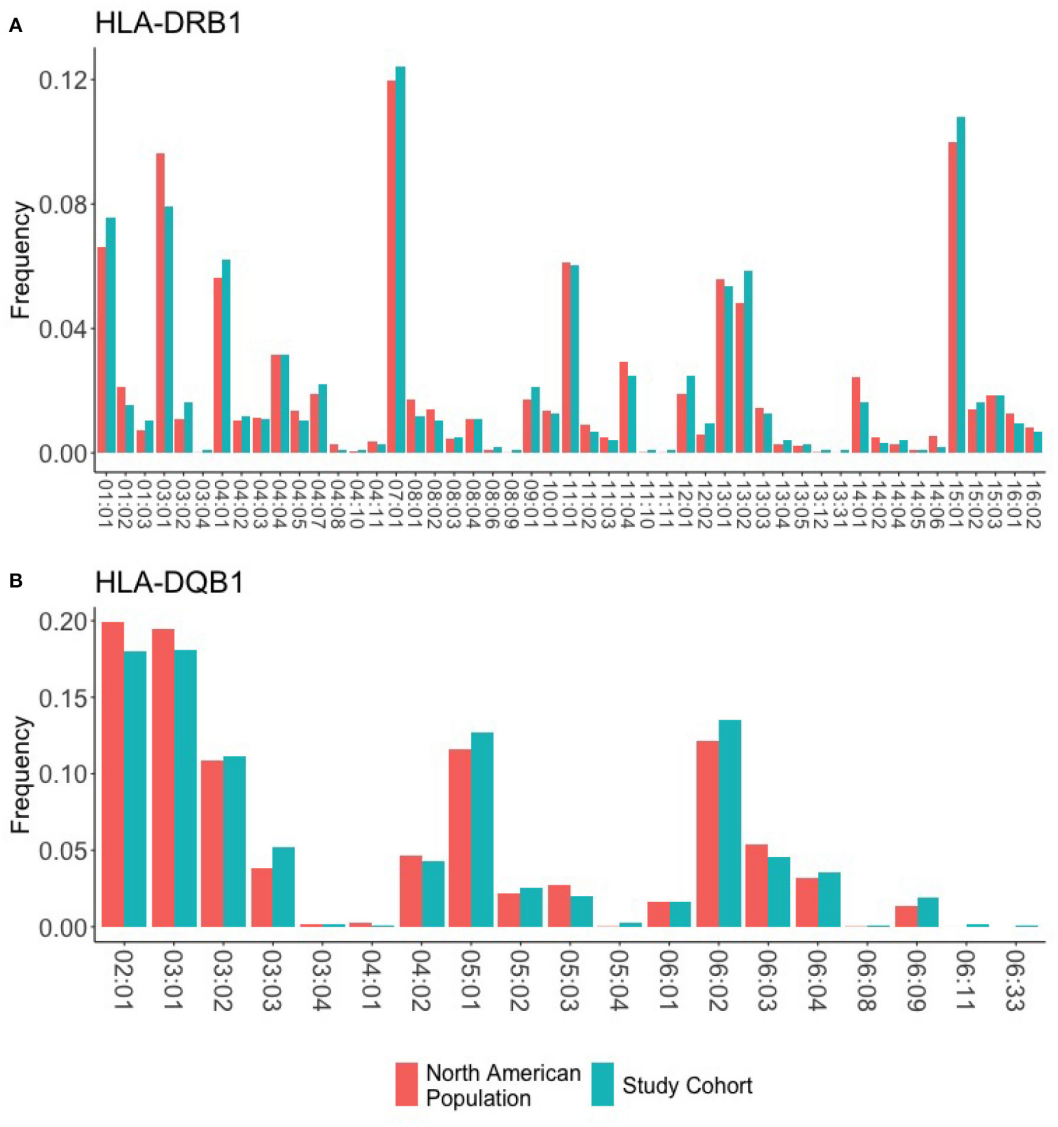
HLA frequencies in the study cohort. HLA frequencies of the 997 participants are compared to the expected frequencies of the North American sub-population. **(A)** The frequencies of HLA-DRB1 alleles in the study cohort (green) closely match the background distribution of alleles in the whole North American population. **(B)** The frequencies of HLA-DQB1 alleles in the study cohort (green) closely match the background distribution of alleles in the whole North American population.

We obtained 1,000 DNA samples from MLOF. The samples were subjected to high resolution (4-digit) HLA typing. The complete data set is presented in Supplementary Table 2. Each HLA variant in our study occurs at a frequency that is comparable to its respective frequency in the North American population (Figure 2). Moreover, the HLA-DRB1 alleles identified in the 997 participants cover 99.5% of the allelic variation in North America. Additionally, 18 alleles representing 82% of the North American population were found at or >30 times. The distribution of HLA-DRB1 and HLA-DQB1 alleles in the cohort is comparable to the distribution of alleles in the North American Population.

### 3.4. Univariate Analysis of Severe HA Participants

Each variable was first analyzed using a univariate logistic regression model (see section 2) and those determined to be significant with a *p*-value of < 0.25 were included in the final multivariate model.

Univariate analysis identified the following HLA alleles (*p* < 0.25) for inclusion in the multivariate model: 9 HLA-DRB1 alleles; HLA-DRB1*01:01, HLA-DRB1*01:03, HLA-DRB1*04:04, HLA-DRB1*04:05, HLA-DRB1*04:07, HLA-DRB1*08:01, HLA-DRB1*11:04, HLA-DRB1*15:01, and HLA-DRB1*15:03; 4 HLA-DQB1 alleles; HLA-DQB1*03:01, HLA-DQB1*05:01, HLA-DQB1*05:02, and HLA-DQB1*06:02; 3 HLA-DPB1 alleles; HLA-DPB1*02:02, HLA-DPB1*18:01, and HLA-DPB1*19:01; As well as HLA-DRB3*01:01 and HLA-DRB5*01:01 (Supplementary Table 3). No HLA-DRB4 alleles met the criterion for inclusion in the multivariate model.

Both Hispanic and Black or African American/Not Hispanic participants, compared to white participants, met the univariate criterion of *p* < 0.25 to be included in the multivariate model (Supplementary Table 4).

Four variant types (compared to intron 22 inversion) were selected for inclusion in the multivariate model: frameshifts, large structural changes (>50 bp), missense variants, and nonsense variants (Supplementary Table 4).

Age, as either a linear predictor or as a third-degree polynomial, was significant for inclusion in the multivariate model. A comparison of log Likelihoods (see section 2); *p* = 0.0094 with 2 degrees of freedom) showed that the third-degree polynomial was a significant addition to the model.

### 3.5. A Multivariate Regression Analysis of Severe HA Participants

**Figure 3:**
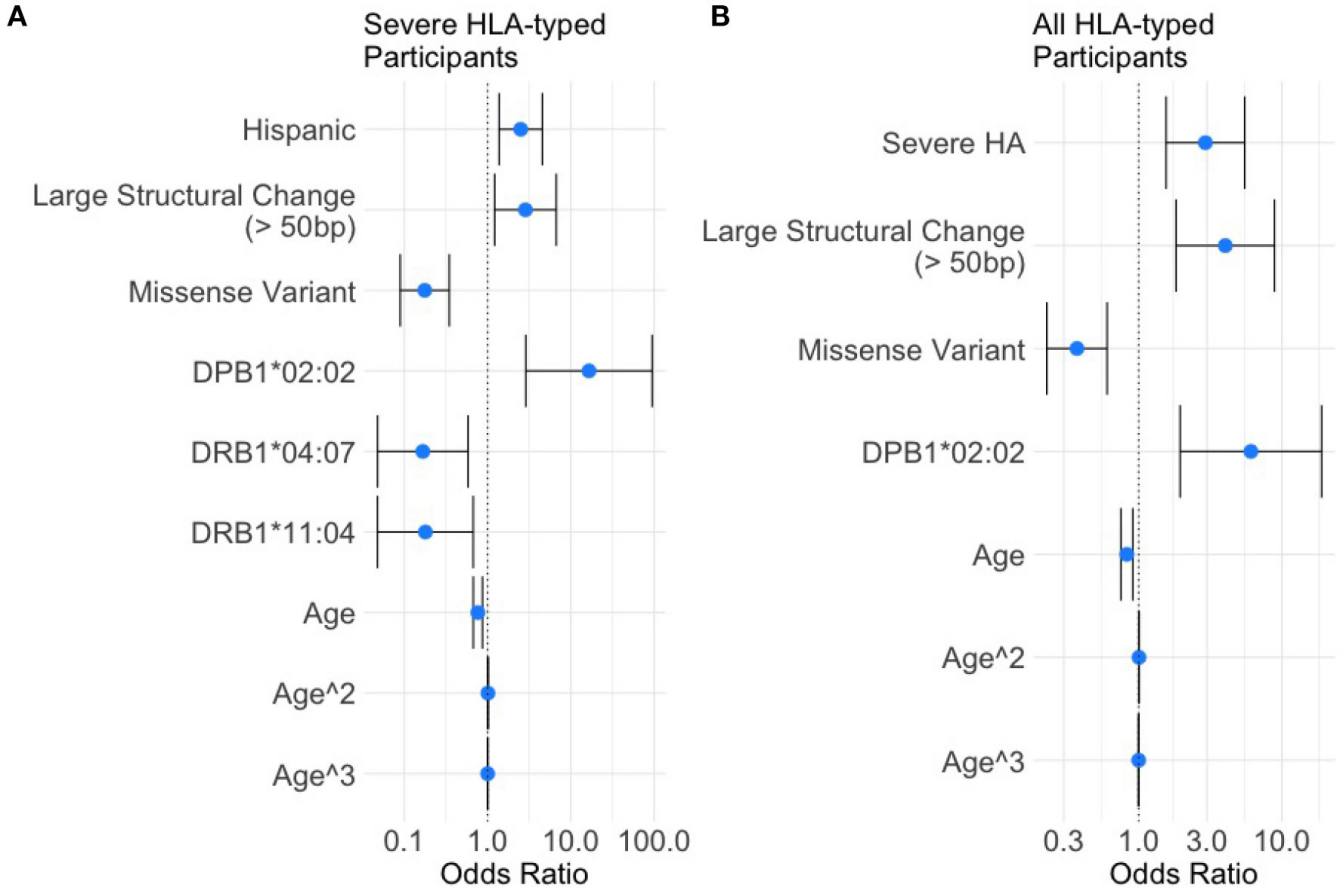
Results of the multivariate analysis. **(A)** Severe HA participants increased odds of inhibitor development were found for Hispanic participants (OR = 2.50, 95%CI 1.374.54), large structural variants (OR = 2.85, 95%CI 1.216.67), and HLA-DPB1*02:02 (OR = 16.50, 95%CI 2.8794.78). Decreased odds were found for missense variants (OR = 0.18, 95%CI 0.0890.35), HLA-DRB1*04:07 (OR = 0.17, 95%CI 0.0480.58), and HLA-DRB1*11:04 (OR = 0.18, 95%CI 0.0480.67). **(B)** All HLA participants increased odds of inhibitor development were found for severe HA (OR = 2.92, 95%CI 1.565.50), Large structural variants (OR = 4.03, 95%CI 1.828.89), and HLA-DPB1*02:02 (OR = 6.08, 95%CI 1.9518.94). Decreased odds were found for missense variants (OR = 0.37, 95%CI 0.220.60).

Based on the variables selected using univariate analysis, a final multivariate model was fit including 18 HLA alleles, one variable for race, four different variant types, and three variables for age (age, age-squared, and age-cubed). *P*-values for the variables were adjusted and results with a false discovery rate of <0.05 were considered significant (Figure 3, Table 4). It was found that controlling for age was extremely important in explaining the higher incidence of inhibitor development in younger participants independent of other factors.

**Table 4:** Multivariate model resultssevere HA participants.

	**Proportion with**	**Odds Ratio**	**Adjusted**	**Significant**
	**inhibitors (%)**	**(95% CI)**	***p*-value**	
**Age**				
Age		0.76 (0.670.87)	4.1e-04	Yes
Age^2^		1.008 (1.0031.012)	3.3e-03	Yes
Age^3^		0.99 (0.990.99)	0.011	Yes
**Race/Ethnicity**				
Black or African				
American	35/83 (42)	1.38 (0.722.65)	0.44	
Non-Hispanic				
Hispanic	39/79 (49)	2.50 (1.374.54)	0.011	Yes
**Variant type**				
Frameshift	30/101 (30)	0.54 (0.310.94)	0.065	
Large structural	21/33 (64)	2.85 (1.216.67)	0.045	Yes
change				
Missense	15/108 (14)	0.18 (0.090.35)	1.4e-5	Yes
Nonsense	32/74 (43)	1.08 (0.611.93)	0.81	
**HLA variant**				
DRB1*01:01	27/90 (30)	0.96 (0.551.69)	0.89	
DRB1*01:03	2/13 (15)	0.30 (0.061.46)	0.29	
DRB1*04:04	18/40 (45)	1.72 (0.803.67)	0.30	
DRB1*04:05	8/14 (57)	2.30 (0.658.16)	0.34	
DRB1*04:07	5/26 (19)	0.17 (0.0480.58)	0.017	Yes
DRB1*08:01	3/15 (20)	0.36 (0.091.44)	0.30	
DRB1*11:04	3/28 (11)	0.18 (0.050.67)	0.033	Yes
DRB1*15:01	55/122 (45)	2.22 (0.2320.90)	0.59	
DRB1*15:03	13/21 (62)	3.26 (0.3431.33)	0.43	
DRB3*01:01	43/144 (30)	0.76 (0.471.23)	0.38	
DRB5*01:01	68/146 (47)	0.38 (0.043.42)	0.49	
DPB1*02:02	8/10 (80)	16.50 (2.8794.78)	9.4e-03	Yes
DPB1*18:01	10/20 (50)	1.20 (0.354.13)	0.81	
DPB1*19:01	6/8 (75)	7.72 (1.3743.55)	0.052	
DQB1*03:01	66/205 (32)	0.91 (0.581.42)	0.75	
DQB1*05:02	14/30 (47)	1.24 (0.473.24)	0.75	
DQB1*05:03	5/23 (22)	0.50 (0.171.47)	0.34	
DQB1*06:02	75/150 (50)	2.03 (0.666.20)	0.34	

One HLA allele was found to be significantly correlated with the increased odds for having developed an inhibitor: HLA-DPB1*02:02 [OR = 16.5, 95% CI (2.87,94.78), adjusted-*p* = 9.35*10^3^].

Two HLA alleles were found to be associated with decreased odds for having developed inhibitors: HLA-DRB1*04:07 [OR = 0.17, 95% CI (0.048, 0.58), adjusted-*p* = 0.0174]; and HLA-DRB1*11:04 [OR = 0.18, 95% CI (0.048, 0.67), adjusted-*p* = 0.0334].

Missense variants were found to be correlated with decreased odds for inhibitor development [OR = 0.18, 95% CI (0.09, 0.35), adjusted-*p* = 1.42*10^5^]. Large structural variants affecting >50 base pairs were associated with increased odds of inhibitor development [OR = 2.85, 95% CI (1.21, 6.67), adjusted-*p* = 0.045].

The effect of age was significant for each of the three variables for age with adjusted-*p* values of 4.1*10^4^, 3.3*10^3^, and 1.1*10^2^. The polynomial allows for a steep decrease in OR from 0 to 20 years, a relatively flat OR from 20 to 60 and slight decreases in OR after age 60. The effect of age is a decreasing odds of inhibitor development generally as participants get older.

### 3.6. Univariate Analysis in All HLA-Typed Participants

Each variable was first analyzed using a univariate logistic regression model (see section 2) and those determined to be significant with a *p*-value of <0.25 were included in the final multivariate model.

Univariate analysis identified the following HLA alleles (*p* < 0.25) for inclusion in the multivariate model: 12 HLA-DRB1 alleles; HLA-DRB1*01:01, HLA-DRB1*01:03, HLA-DRB1*04:04, HLA-DRB1*04:05, HLA-DRB1*04:07, HLA-DRB1*08:01, HLA-DRB1*11:01, HLA-DRB1*11:04, HLA-DRB1*12:01, HLA-DRB1*12:02, HLA-DRB1*15:01, and HLA-DRB1*15:03; 4 HLA-DQB1 alleles; HLA-DQB1*03:01, HLA-DQB1*05:01, HLA-DQB1*05:02, and HLA-DQB1*06:02; 7 HLA-DPB1 alleles; HLA-DPB1*02:02, HLA-DPB1*03:01, HLA-DPB1*05:01, HLA-DPB1*10:01, HLA-DPB1*11:01, HLA-DPB1*18:01, and HLA-DPB1*19:01; and HLA-DRB3*01:01 and HLA-DRB5*01:01 (Supplementary Table 5). No HLA-DRB4 alleles met the criterion for inclusion in the multivariate model.

Both severe HA and moderate HA diagnoses, as compared to mild HA, met the criterion of a *p* < 0.25 for inclusion into the multivariate model (Supplementary Table 6).

Similarly, only one race/ethnicity (compared to White), Black or African American/Not Hispanic, met the univariate criterion of *p* < 0.25 to be included in the multivariate model (Supplementary Table 6).

Four variant types (compared to intron 22 inversion) were selected for inclusion in the multivariate model: intron 1 inversions, large structural changes (>50 bp), missense variants, and nonsense variants (Supplementary Table 6).

Age, as either a linear predictor or as a third-degree polynomial, was significant for inclusion in the multivariate model. A comparison of log Likelihoods (see section 2; *p* = 0.0105 with 2 degrees of freedom) showed that the third-degree polynomial was a significant addition to the model.

### 3.7. A Multivariate Regression Analysis of All HLA-Typed Participants

Based on the variables selected using univariate analysis, a final multivariate model was fit including 25 HLA alleles, one variable for race/ethnicity, four different variant types, two variables for disease severity, and three variables for age (age, age-squared, and age-cubed). *P*-values for the variables were adjusted and results with a false discovery rate of <0.05 were considered significant (Figure 3, Table 5).

**Table 5:** Multivariate model resultsall HLA-typed participants.

	**Proportion with**	**Odds Ratio**	**Adjusted**	**Significant**
	**Inhibitors (%)**	**(95% CI)*p*-value**		
**Age**				
Age		0.83 (0.750.91)	0.0012	Yes
Age^2^		1.01 (1.0021.008)	0.007	Yes
Age^3^		0.99 (0.990.99)	0.023	Yes
**Race/Ethnicity**				
Black or African				
American	40/106 (38)	1.14 (0.632.06)	0.79	
non-hispanic				
**Variant type**				
Intron 1 inversion	7/16 (44)	2.28 (0.687.66)	0.28	
Large structural	22/35 (63)	4.02 (1.828.89)	0.007	Yes
change				
Missense	45/439 (10)	0.37 (0.230.60)	0.002	Yes
Nonsense	32/76 (42)	1.51 (0.882.59)	0.23	
***Hemophilia Severity***				
Moderate	17/36 (12)	1.41 (0.662.99)	0.47	
Severe	217/612 (35)	2.92 (1.565.50)	0.008	Yes
***HLA Variant***				
DRB1*01:01	31/150 (21)	0.88 (0.421.69)	0.82	
DRB1*01:03	2/20 (10)	0.22 (0.041.18)	0.20	
DRB1*04:04	24/68 (35)	2.03 (1.103.79)	0.08	
DRB1*04:05	9/19 (47)	2.32 (0.776.97)	0.23	
DRB1*04:07	5/45 (11)	0.27 (0.090.82)	0.08	
DRB1*08:01	5/31 (16)	0.60 (0.211.76)	0.47	
DRB1*11:01	38/122 (31)	1.90 (1.043.46)	0.11	
DRB1*11:04	5/56 (9)	0.39 (0.131.13)	0.20	
DRB1*12:01	14/41 (34)	2.22 (0.955.22)	0.19	
DRB1*12:02	5/11 (45)	1.57 (0.396.37)	0.66	
DRB1*15:01	61/204 (30)	4.43 (0.4430.56)	0.23	
DRB1*15:03	16/30 (53)	9.85 (1.3671.03)	0.08	
DRB3*01:01	48/240 (20)	0.71 (0.471.08)	0.23	
DRB5*01:01	76/237 (32)	0.24 (0.041.63)	0.24	
DPB1*02:02	9/18 (50)	6.08 (1.9518.94)	0.011	Yes
DPB1*03:01	40/185 (22)	0.80 (0.511.26)	0.47	
DPB1*05:01	20/62 (32)	0.91 (0.471.79)	0.84	
DPB1*10:01	11/30 (37)	1.96 (0.824.71)	0.23	
DPB1*11:01	9/53 (17)	0.58 (0.251.34)	0.30	
DPB1*18:01	12/26 (46)	0.99 (0.333.03)	0.99	
DPB1*19:01	7/14 (50)	4.14 (1.2214.03)	0.08	
DQB1*03:01	75/345 (22)	0.66 (0.411.06)	0.20	
DQB1*05:01	54/242 (22)	1.03 (0.551.93)	0.96	
DQB1*05:02	16/38 (42)	1.72 (0.734.06)	0.31	
DQB1*06:02	83/241 (34)	1.21 (0.463.19)	0.82	

Severe HA participants had a higher rate of inhibitor formation [OR = 2.92, 95% CI (1.56, 5.50), adjusted-*p* = 0.0075].

One HLA allele was found to be associated with decreased odds for inhibitor development: HLA-DPB1*02:02 [OR = 6.08, 95% CI (1.95, 18.94), adjusted-*p* = 0.011]. No HLA alleles were found to be significantly correlated with decreased odds for having developed an inhibitor.

Missense variants were found to be correlated with decreased odds for inhibitor development [OR = 0.37, 95% CI (0.23, 0.60), adjusted-*p* = 0.0018]. Large structural variants affecting greater than 50 base pairs were associated with increased odds of inhibitor development [OR = 4.02, 95% CI (1.82, 8.89), adjusted-*p* = 0.0067].

The effect of age was significant for each of the three variables for age with adjusted-*p* values of 0.0012, 0.007, and 0.023. The polynomial allows for a steep decrease in OR from 0 to 20 years, a relatively flat OR from 20 to 60 and slight decreases in OR after age 60. The effect of age is a decreasing odds of inhibitor development generally as participants get older.

### 3.8. Comparison of Predicted Binding Affinities of Foreign Sequences at the Location of Missense Mutation in the *F8* Gene of Study Participants

The median percentile rank binding affinity in participants who had ever had an inhibitory response was 5.5 as compared to 7.5 for participants who had never had an inhibitory response. As the data was found to be non-Gaussian even after transformations were applied (Shapiro-Wilk *p*-values of 7.67 * 10^7^ and 2.2* 10^16^, respectively) the non-parametric MannWhitney *U*-Test was used. This test rejected to null hypothesis that the distributions of the two samples was similar (Supplementary Figure 1).

## 4. Discussion

Published work supports the postulate that genetic factors play an important role in the development of inhibitors to FVIII drug products (4, 5, 10,14, 19). A meta-analysis published in 2012 showed that *F8* variant type influenced inhibitor development in HA patients (5). In the study reported here involving 612 participants with severe HA who were HLA typed, we found that the risk of inhibitor development was higher in participants with large (>50 bp) structural variants (OR = 2.85) and lower in patients with missense variants (OR = 0.18; Figure 3, Table 4) which is consistent with the meta-analysis of previous studies. However, other genetic risk factors for inhibitor development have not been researched to the same extent as *F8* variants. One of the most important genetic variables associated with immune responses is the HLA repertoire. The handful of studies on the association between specific HLAs and inhibitors (10,14) involve 57 to 176 participants which is too low for making statistical estimates.

In this survey we have focused on the HLA-DRB1/3/4/5, HLA-DPB1, and HLA-DQB1 genes. The generation of anti-drug antibodies to replacement proteins is driven by CD4+ helper T cells (9). This pathway involves the HLA Class II molecules. The HLA-DRB1 variants are the most diverse Class II molecules and are predominantly, but not exclusively, involved in the presentation of peptides derived from protein drugs. For instance, in a recent study of FVIII peptides identified on monocyte-derived dendritic cells, 78% of the peptides were found to bind HLA-DRB1 (32).

The MLOF data set includes pathogenic *F8* variant data for 7,151 participants. All samples were processed at a central facility using the same validated method (33). It was not cost-effective for us to HLA type all 7,151 participants. We determined through simulation that 1,000 participants were expected to provide adequate coverage of alleles while also having enough samples of common alleles to allow for statistical comparisons.

We obtained high-resolution HLA typing for 997 of the 1,000 participants. The HLA-DRB1 alleles identified represent 99% of the allelic variation in North America. The individual HLA-DRB1 and HLA-DPQ1 alleles in our data set occur at frequencies comparable to those found in the North American population (Figure 2).

Based on ORs in our analysis of participants with severe HA from a multivariate binomial logistic regression, we determined that only one HLA variant, DPB1*02:02, was associated with a higher risk of inhibitor development with an OR of 16.50 (95% CI 2.8794.78). Two variants, DRB1*04:07 and DRB1*11:04, were associated with a lower risk of inhibitor development with ORs of 0.17 (95% CI 0.0480.58) and 0.18 (95% CI 0.050.67; Figure 3, Table 4). While similar trends were found in the data examining all HLA-typed participants, the two seemingly protective HLA-DRB1 variants failed to reach statistical significance (Figure 3, Table 5).

Several studies indicate that Hispanic HA patients are at higher risk of developing inhibitors than White patients (34,36). Using the 612 HLA typed participants with severe HA from the MLOF Research Repository provided similar results. Hispanic participants had an association with higher rates of inhibitor formation (OR = 2.50, 95% CI 1.374.54; Figure 3, Table 4). The differential inhibitor-risk based on race independent of HLA-type is interesting in the context of HLA repertoires because human sub-populations have different relative frequencies of HLA variants (37).

An interesting aspect of our findings is that HLA variants identified as having a significant association with inhibitor development are relatively rare in the North American population. Moreover, the HLA-DPB1*02:02 allele occurred in only 10 participants which is less frequent than most other alleles. It is only because we HLA typed a relatively large cohort of participants that these rare HLA alleles were identified in sufficient numbers to obtain statistical significance in multivariate analyses.

Our analysis failed to confirm findings in previous works comparing HLA-type to inhibitor development (3, 7, 10,14). Plausible reasons include the small sample sizes in previous studies and the reliance on univariate analyses failing to control for correlation with other cofactors. It is not possible to determine if these studies included a representative distribution of HLA variants or sufficient numbers of replicates of each variant for statistical analysis. However, given the limited number of participants in each study, 57176, it is unlikely that these criteria were met.

A limitation of our study is the possibility of bias in the selection of participants for HLA-typing. As this dataset involved a *post-hoc* analysis of data, the data was not collected with this study in mind. Incomplete clinical data and available DNA for HLA-testing forced us to select for participants who could satisfy the needs of this study. Our study is an important step in identifying correlates with significant effects of a patient's risk of inhibitor development. We hope that this will help to inform future research on the relationship between HLA-type (independently and in association with other genetic markers) and inhibitor positive patients with HA.

To illustrate this class of studies, we used the subset of participants with a missense mutation in the *F8* gene to explore the hypothesis that foreign-peptide-HLA-DRB1 binding affinity is a risk factor for inhibitor development. Based on inhibitor data from HA participants with missense mutations, several previous studies support this hypothesis (7, 38, 39). The hypothesis is based on the rationale that presentation of foreign peptides is an initial, necessary step in eliciting an immune response to a protein therapeutic (9). Thus, for an immune response to be elicited two conditions must be met; (a) the infused protein must generate peptides that are foreign to the patient and (b) these foreign peptides must be efficiently presented to the immune system by HLA molecules. Foreign peptides that bind with high affinity to an individual patient's HLA with high affinity have a lower off-rate and thus a higher probability of eliciting an immune response. The median percentile rank binding affinity in participants who were inhibitor positive was 5.5 as compared to 7.5 for participants who had never developed inhibitors. As the data was found to be non-Gaussian even after transformations were applied (ShapiroWilk *p*-values of 7.67* 10^7^ and 2.2* 10^16^, respectively), the non-parametric MannWhitney *U*-Test was used. This test rejected to null hypothesis that the distributions of the two samples was similar (Supplementary Figure 1).

In this study we present a data set of 997 fully HLA-typed participants with HA. The HLA typed subset is derived from a larger set of 7,151 participants. The 997 HLA-typed participants capture the heterogeneity of the HA participant population with respect to *F8* variants, severity of disease and racial diversity. Moreover, the HLA-DRB1 variants identified represent 98% of the North American Population, occur at relative frequencies observed in the wider population and include sufficient replicates of each HLA-DRB1 variant for meaningful statistical analyses. Using this data set we identified 1 HLA variant associated with an increased risk of inhibitors and 2 HLA variants associated with a reduced risk of inhibitors. The MLOF Research Repository is an extremely useful data set for uncovering the genetic determinants associated with inhibitor development in HA. The HLA repertoire represents an important and highly variable genetic characteristic of HA patients that was lacking. The enhanced data set can now be used to generate more complex models to identify biomarkers for predicting inhibitor development in HA patients.

## Data Availability Statement

The original contributions presented in the study are included in the article/Supplementary Material, further inquiries can be directed to the corresponding author/s.

## Ethics Statement

The studies involving human participants were reviewed and approved by Western International Review Board. Written informed consent to participate in this study was provided by the participants' legal guardian/next of kin.

## Author Contributions

JM and ZS designed the research. JM and VS performed the research. and JM, VS, and ZS wrote the paper. All authors contributed to the article and approved the submitted version.

## Conflict of Interest

The authors declare that the research was conducted in the absence of any commercial or financial relationships that could be construed as a potential conflict of interest.
