# Supplementary material for: HLA Variants and Inhibitor Development in Hemophilia A: A Retrospective Case-Controlled Study Using the ATHNdataset
Source: Front Med (Lausanne). 2021 May 7;8:663396. doi: 10.3389/fmed.2021.663396 (PMC8139405; doi:10.3389/fmed.2021.663396)
Supplement: Supplementary file 1 [file Data_Sheet_1.pdf]

Supplementary Material

1 SUPPLEMENTARY TABLES AND FIGURES

1.1 Figures

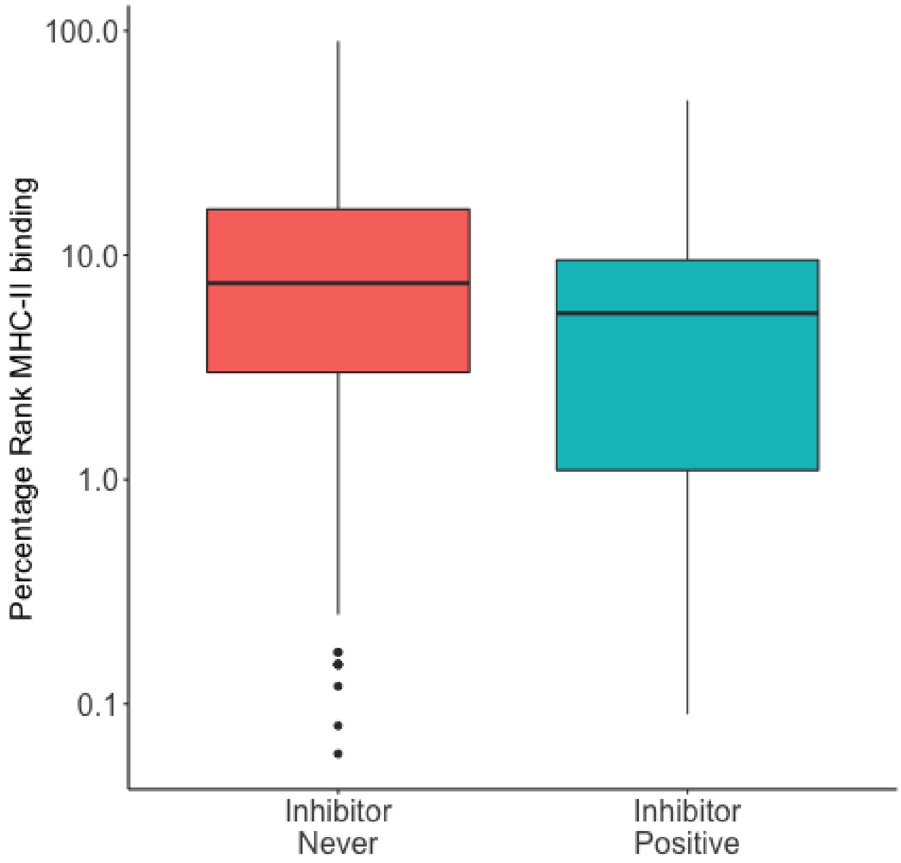

Figure S1. Predicted Binding Affinities as defined by percentage rank *see Methods*. Inhibitor positive participants had a lower percentage rank binding affinity (median=5.5) than participants with no inhibitor ever (median=7.5). This difference was significant using a one-sided Mann-Whitney U Test (p=0.0225)

1.2 Tables

Table S1: Cohort Selected for HLA-typing as compared to the entire ATHNdataset

	HLA Typed Cohort		Entire ATHNdataset	
	Count	Percentage	Count	Percentage
Disease Severity				
Mild	249	24.97	2310	32.30
Moderate	136	13.64	1163	16.26
Severe	612	61.38	3678	51.43
Primary Treatment Type				
Episodic	1665	27.62	175	15.58
Immune Tolerance Induction	6	0.10	36	3.21
Prophylaxis	2065	34.26	555	49.42
Unknown	2292	38.02	357	31.79
Race/Ethnicity				
American Indian or Alaska Native	7	0.70	7ss7	1.08
Asian	43	4.31	284	3.97
Black or African American Non-Hispanic	106	10.63	709	9.91
Hispanic	141	14.14	1236	17.28
Mixed Race	9	0.90	70	0.98
Native Hawaiian or Other Pacific Islander	3	0.30	24	0.24
White Non-Hispanic	682	68.41	4668	65.28
None Reported	6	0.60	83	1.16
Age (years)				
< 5	19	1.91	461	6.45
5-14	263	26.38	1802	25.20
15-24	273	27.38	1706	23.86
25-39	239	23.97	1639	22.92
40-64	155	15.55	1178	16.47
$\geq$65	48	4.81	365	5.10
Variant Type				
5’ Upstream	2	0.20	11	0.15
Frameshift	106	10.63	603	8.43
Intron 1 Inversion	16	1.60	65	0.91
Intron 22 Inversion	267	26.78	1529	21.38
Large Structural Change[1]	35	3.51	233	3.26
Small Structutral Change[2]	5	0.50	35	0.49
Missense	439	44.03	2757	38.55
Nonsense	76	7.62	409	5.72
Splice Site Change	23	2.31	184	2.57
Synonymous	25	2.51	174	2.43
Untranslated Region	0	0.00	2	0.03
None Reported	3	0.30	1149	16.07

Table S2: HLA-Typing Results

	DRB11	DRB12	DRB31	DRB32	DRB41	DRB42	DRB51	DRB52	DQB11	DQB12	DPB11	DPB12
23RGM7XKF	14:01	14:01	02:02:01G	-	-	-	-	-	05:03	05:03	01:01	03:01
23Z9AXSRK	04:01	11:04	02:02:01G	-	01:01:01G	-	-	-	03:01	03:01	04:01	06:01
26TN4QSH9	13:01	15:01	02:02:01G	-	-	-	01:01:01G	-	06:02	06:03	04:01	13:01
2ADXZPU4Y	04:11	14:04	02:02:01G	-	01:01:01G	-	-	-	03:02	05:03	03:01	27:01
2BHKmPgV4	01:01	07:01	-	-	01:01:01G	-	-	-	02:01	05:01	11:01	18:01
2BSZRF3D8	07:01	11:04	02:02:01G	-	01:01:01G	-	-	-	03:01	03:03	04:01	04:02
2CY36RKA8	13:01	15:01	02:02:01G	-	-	-	01:01:01G	-	06:02	06:03	13:01	17:01
2EDXNVTP9	04:07	07:01	-	-	01:01:01G	-	-	-	02:01	03:02	04:01	04:02
2FV6QPTRH	11:01	12:01	02:02:01G	-	-	-	-	-	05:01	06:02	18:01	19:01
2GNT4WKXU	04:07	09:01	-	-	01:01:01G	-	-	-	03:01	03:03	03:01	03:01
2KQ5PJ7B3	01:02	07:01	-	-	01:01:01G	-	-	-	03:03	05:01	01:01	04:01
2N6UTBXWY	03:01	11:01	01:01:02G	02:02:01G	-	-	-	-	02:01	03:01	01:01	03:01
2NJAFK5SY	04:04	13:03	01:01:02G	-	01:01:01G	-	-	-	03:01	03:02	02:01	04:02
2QU45YP9F	03:01	15:01	01:01:02G	-	-	-	01:01:01G	-	02:01	06:02	02:01	04:01
2S95QDKGF	01:01	01:01	-	-	-	-	-	-	05:01	05:01	04:02	14:01
2SGFBRCK6	08:02	11:02	02:02:01G	-	-	-	-	-	03:01	04:02	04:01	04:02
2ST85WPKX	13:02	15:01	03:01:01G	-	-	-	01:01:01G	-	06:02	06:04	02:01	04:01
2UPX6MWFJ	04:02	04:07	-	-	01:01:01G	-	-	-	03:02	03:02	02:01	04:02
2UREMNGSD	12:02	15:02	03:01:01G	-	-	-	01:01:01G	-	05:02	05:02	01:01	05:01
2VWMSNCKH	01:02	03:01	01:01:02G	-	-	-	-	-	02:01	05:01	02:01	13:01
2XUE96HSY	10:01	15:03	-	-	-	-	01:01:01G	-	05:01	06:02	02:01	10:01
2YWA6MUVC	04:01	14:01	02:02:01G	-	01:01:01G	-	-	-	03:01	05:03	03:01	04:01
2zc6uhbsm	13:01	15:01	01:01:02G	-	-	-	01:01:01G	-	06:02	06:03	04:02	05:01
2ZSUMCYP5	04:07	04:07	-	-	01:01:01G	-	-	-	03:02	03:02	03:01	04:02
345EV6KXF	03:01	04:03	01:01:02G	-	01:01:01G	-	-	-	02:01	03:02	04:01	04:01
34EUDY8RK	07:01	13:03	01:01:02G	-	01:01:01G	-	-	-	03:01	03:03	02:01	03:01
3598VZUMN	03:01	03:01	01:01:02G	-	-	-	-	-	02:01	02:01	04:01	04:01
365AJCSYH	03:01	14:02	01:01:02G	02:02:01G	-	-	-	-	02:01	03:01	02:02	03:01
36DMYSX2H	08:01	08:02	-	-	-	-	-	-	04:02	04:02	04:01	04:01
3aj6u42Rs	01:02	15:01	-	-	-	-	01:01:01G	-	05:01	06:01	01:01	09:01
3BKEAWH7N	11:01	13:01	02:02:01G	-	-	-	-	-	03:01	06:03	01:01	03:01
3BUEYK2GP	04:01	07:01	-	-	01:01:01G	-	-	-	03:02	03:03	04:01	16:01
3DBH8R7MG	01:01	15:01	-	-	-	-	01:01:01G	-	05:01	06:02	04:01	15:01
3E5WAUVGR	11:01	13:03	01:01:02G	02:02:01G	-	-	-	-	03:01	03:01	02:01	16:01
3EK5WYNBV	03:02	13:02	01:01:02G	03:01:01G	-	-	-	-	04:02	06:09	01:01	01:01
3ER59X46S	04:02	11:01	02:02:01G	-	01:01:01G	-	-	-	03:01	03:02	02:01	04:01
3F26RBUKG	10:01	15:03	-	-	-	-	01:01:01G	-	05:01	06:02	01:01	01:01
3FQPMXYZC	13:01	13:02	01:01:02G	03:01:01G	-	-	-	-	06:03	06:04	04:01	04:02
3FSGKN2R4	01:02	07:01	-	-	01:01:01G	-	-	-	02:01	05:01	01:01	85:01
3GWTEX94S	01:01	15:01	-	-	-	-	01:01:01G	-	05:01	06:02	04:01	04:02
3HPKYRTAQ	04:01	15:01	-	-	01:01:01G	-	01:01:01G	-	03:01	06:02	04:01	04:01
3J8KZER2X	03:01	03:01	01:01:02G	-	-	-	-	-	02:01	02:01	04:01	17:01
3M4TPN76Y	03:01	04:03	01:01:02G	-	01:01:01G	-	-	-	02:01	03:02	01:01	04:01
3M7W58ZGY	01:01	13:02	03:01:01G	-	-	-	-	-	05:01	06:04	02:01	04:01
3NQECH4SJ	03:01	15:01	01:01:02G	-	-	-	01:01:01G	-	02:01	06:02	04:01	04:01
3QFKSDZ9Y	15:03	16:02	-	-	-	-	01:01:01G	02:02:01G	05:02	06:02	13:01	85:01
3TMQDYCXK	13:02	13:02	03:01:01G	-	-	-	-	-	06:04	06:09	02:01	04:01
3UJWXZRVN	11:04	13:02	02:02:01G	03:01:01G	-	-	-	-	03:01	06:09	04:02	05:01
3VBERGUXK	04:01	15:03	-	-	01:01:01G	-	01:01:01G	-	03:02	06:02	04:02	18:01
3WRHZX9TC	07:01	11:01	02:02:01G	-	01:01:01G	-	-	-	03:01	03:03	04:01	13:01
3XBER68W9	03:01	13:02	01:01:02G	03:01:01G	-	-	-	-	02:01	06:04	03:01	04:01
3YBR7NGED	11:01	15:01	02:02:01G	-	-	-	01:01:01G	-	03:01	06:02	04:01	04:01
428KRX7U5	03:01	07:01	01:01:02G	-	01:01:01G	-	-	-	02:01	02:01	01:01	14:01
43azmv5rT	08:04	15:01	-	-	-	-	01:01:01G	-	04:02	06:02	02:01	03:01
45UKVPQGA	04:04	08:01	-	-	01:01:01G	-	-	-	03:02	04:02	04:01	06:01

463AQ9VKW	11:01	13:01	01:01:02G	02:02:01G	-	-	-	-	03:01	06:03	04:01	04:01
46E9FQ5XM	08:03	10:01	-	-	-	-	-	-	05:01	06:01	03:01	21:01
46Q8ETKJP	04:07	07:01	-	-	01:01:01G	-	-	-	03:01	03:03	03:01	04:02
47ZRCU5MP	07:01	13:01	02:02:01G	-	01:01:01G	-	-	-	02:01	06:03	04:01	04:01
48DVFBCWS	01:02	03:01	02:02:01G	-	-	-	-	-	02:01	05:01	02:01	04:01
498UNH36Y	04:07	08:02	-	-	01:01:01G	-	-	-	03:02	04:02	04:02	04:02
49X5EMC8A	15:01	15:03	-	-	-	-	01:01:01G	-	06:02	06:02	01:01	04:01
4A7NTM3Q5	01:01	13:02	03:01:01G	-	-	-	-	-	05:01	06:04	03:01	04:02
4B8ECGA5U	01:01	13:01	02:02:01G	-	-	-	-	-	05:01	06:03	02:01	04:01
4DQZKNREV	04:01	11:01	02:02:01G	-	01:01:01G	-	-	-	03:01	03:02	02:01	15:01
4DVBKHCXG	07:01	07:01	-	-	01:01:01G	-	-	-	02:01	02:01	03:01	11:01
4EAZ6YSCN	04:03	11:01	02:02:01G	-	01:01:01G	-	-	-	03:01	03:02	04:01	04:01
4GVCD59BP	03:01	04:04	01:01:02G	-	01:01:01G	-	-	-	02:01	03:02	01:01	06:01
4HFU5PN2Y	04:01	13:01	01:01:02G	-	01:01:01G	-	-	-	03:01	06:03	04:01	04:02
4JWNMZGCU	13:01	15:01	01:01:02G	-	-	-	01:01:01G	-	06:02	06:03	02:01	04:01
4MQAF8PKJ	01:03	13:02	03:01:01G	-	-	-	-	-	05:01	06:04	04:01	10:01
4MSTCFG2Z	01:01	09:01	-	-	01:01:01G	-	-	-	03:03	05:01	02:01	04:01
4PV5CXY8W	04:01	04:07	-	-	01:01:01G	-	-	-	03:01	03:02	02:01	17:01
4Q6hX9S8F	13:01	15:01	01:01:02G	-	-	-	01:01:01G	-	06:02	06:03	03:01	20:01
4QMYTHV6X	03:01	04:04	02:02:01G	-	01:01:01G	-	-	-	02:01	03:02	02:01	04:02
4QY9SBE68	03:01	11:01	01:01:02G	02:02:01G	-	-	-	-	02:01	03:01	01:01	04:01
4SDKYXFTE	04:04	14:01	02:02:01G	-	01:01:01G	-	-	-	03:02	05:03	04:01	04:02
4SRD9HC7U	11:04	15:01	02:02:01G	-	-	-	01:01:01G	-	03:01	06:02	04:01	04:02
4SWRP2CTY	08:03	15:02	-	-	-	-	01:02:01G	-	05:01	06:01	02:02	05:01
4T3ENUDZ8	08:02	08:02	-	-	-	-	-	-	04:02	04:02	04:02	04:02
4UDCYA7Q3	04:02	07:01	-	-	01:01:01G	-	-	-	02:01	03:02	03:01	04:01
4WRFS98XH	07:01	08:02	-	-	01:01:01G	-	-	-	02:01	04:02	04:02	11:01
4WUMYAFXQ	07:01	07:01	-	-	01:01:01G	-	-	-	02:01	02:01	04:02	17:01
4WVFECPDB	04:01	13:02	03:01:01G	-	01:01:01G	-	-	-	03:02	06:04	04:01	04:01
4XQPA5GB7	03:01	10:01	01:01:02G	-	-	-	-	-	02:01	05:01	04:01	17:01
4ZGM9KR7C	07:01	07:01	-	-	01:01:01G	-	-	-	02:01	02:01	04:01	14:01
53FN79QBH	03:01	15:01	02:02:01G	-	-	-	01:01:01G	-	02:01	06:02	04:01	13:01
53PN8FYMB	03:01	15:02	02:02:01G	-	-	-	01:02:01G	-	02:01	06:01	02:01	02:02
54UDQWCER	08:01	15:01	-	-	-	-	01:01:01G	-	04:02	06:02	04:01	04:01
56PDJAWU3	04:01	07:01	-	-	01:01:01G	-	-	-	03:02	03:03	04:01	04:01
57S8UFRA6	01:01	15:01	-	-	-	-	01:01:01G	-	05:01	06:02	03:01	04:01
58JN94FH3	07:01	08:01	-	-	01:01:01G	-	-	-	03:03	04:02	03:01	04:01
59RQJTH7G	01:01	13:02	03:01:01G	-	-	-	-	-	05:01	06:09	04:01	04:02
5BAMYRNWD	03:01	04:08	01:01:02G	-	01:01:01G	-	-	-	02:01	03:01	02:02	04:01
5BV9HS3TR	01:01	07:01	-	-	01:01:01G	-	-	-	03:03	05:01	04:01	04:02
5BZJNC7SW	01:01	04:01	-	-	01:01:01G	-	-	-	03:02	05:01	03:01	04:01
5EKFMS6BJ	09:01	15:01	-	-	01:01:01G	-	01:01:01G	-	03:03	06:02	04:01	04:02
5EP3J7HZG	08:01	13:01	02:02:01G	-	-	-	-	-	04:02	06:03	04:02	13:01
5FZGQX6P4	07:01	15:01	-	-	01:01:01G	-	01:01:01G	-	02:01	06:02	04:01	04:02
5GAXVJMQR	07:01	13:03	01:01:02G	-	01:01:01G	-	-	-	02:01	03:01	02:01	03:01
5HEC2X8SN	01:03	07:01	-	-	01:01:01G	-	-	-	02:01	05:01	04:01	11:01
5KSFCYUGQ	07:01	12:01	02:02:01G	-	01:01:01G	-	-	-	02:01	03:01	04:02	16:01
5KX9Y82SZ	04:01	15:01	-	-	01:01:01G	-	01:01:01G	-	03:02	06:02	04:01	14:01
5MGDH84FY	01:01	07:01	-	-	01:01:01G	-	-	-	03:03	05:01	01:01	04:01
5MPQ9BDKH	01:01	13:02	03:01:01G	-	-	-	-	-	05:01	05:01	02:01	04:01
5MYFV826C	11:04	14:01	02:02:01G	-	-	-	-	-	03:01	05:03	04:01	16:01
5NSBR4U2J	01:01	07:01	-	-	01:01:01G	-	-	-	02:01	05:01	02:01	04:02
5NX84WH3Z	13:02	16:01	03:01:01G	-	-	-	02:02:01G	-	05:02	06:09	02:01	13:01
5p8e9XzGU	07:01	16:02	-	-	01:01:01G	-	02:02:01G	-	02:01	03:01	04:01	04:02
5Q2HPMBKY	07:01	14:02	01:01:02G	-	01:01:01G	-	-	-	02:01	03:01	04:01	04:02
5QC7FVTGE	01:01	04:01	-	-	01:01:01G	-	-	-	03:02	05:01	01:01	04:01
5R7YTASC8	03:01	12:01	01:01:02G	02:02:01G	-	-	-	-	02:01	03:01	03:01	04:01

5S7FG9AMK	03:01	15:01	01:01:02G	-	-	-	01:01:01G	-	02:01	06:02	01:01	23:01
5TQCB632S	01:01	07:01	-	-	01:01:01G	-	-	-	02:01	05:01	02:01	04:02
5V6KPJU83	04:03	15:01	-	-	01:03:03	-	01:01:01G	-	03:04	06:02	04:02	04:02
5WE9NMF6Z	11:10	15:03	02:02:01G	-	-	-	01:01:01G	-	06:02	06:02	01:01	17:01
5WNKBVRYC	13:01	15:01	01:01:02G	-	-	-	01:01:01G	-	06:02	06:03	04:01	04:01
5WVGDNEB2	01:01	03:01	02:01:01G	-	-	-	-	-	02:01	05:01	02:01	03:01
5YEVKFBQU	07:01	08:04	-	-	01:01:01G	-	-	-	03:01	03:03	02:01	04:01
5YSMVKFE9	04:02	07:01	-	-	01:01:01G	-	-	-	02:01	03:02	05:01	11:01
5Z8QEVNSJ	14:01	15:01	02:02:01G	-	-	-	01:01:01G	-	05:03	06:02	04:02	10:01
5ZVC8YU3K	03:01	08:01	01:01:02G	-	-	-	-	-	02:01	04:02	03:01	04:02
63ANM5B4X	07:01	15:01	-	-	01:01:01G	-	01:01:01G	-	02:01	06:02	04:01	17:01
64SM5T3PH	01:01	09:01	-	-	01:01:01G	-	-	-	03:03	05:01	04:02	16:01
67KJT3DPU	03:01	03:01	01:01:02G	-	-	-	-	-	02:01	06:02	04:01	04:01
687S5423J	03:01	03:01	01:01:02G	02:02:01G	-	-	-	-	02:01	02:01	01:01	04:01
6BG79XHR5	15:01	16:02	-	-	-	-	01:01:01G	02:02:01G	03:01	06:02	02:01	10:01
6BR8S7Z4W	04:01	13:01	01:01:02G	-	01:01:01G	-	-	-	03:01	06:03	04:01	23:01
6DEY5THN4	01:01	15:01	-	-	-	-	01:01:01G	-	05:01	06:02	04:01	04:01
6DJK4C9E8	03:02	07:01	01:01:02G	-	01:01:01G	-	-	-	02:01	04:02	01:01	13:01
6ERHVNDUX	07:01	11:01	02:02:01G	-	01:01:01G	-	-	-	02:01	03:01	11:01	14:01
6FB4CXSVD	01:01	13:02	03:01:01G	-	-	-	-	-	05:01	06:04	04:02	04:02
6G4NBMKQE	03:01	04:02	01:01:02G	-	01:01:01G	-	-	-	02:01	03:02	01:01	02:01
6GNVSMWX4	08:06	15:01	-	-	-	-	01:01:01G	-	06:01	06:02	01:01	04:01
6GT8VBEQH	01:03	13:02	03:01:01G	-	-	-	-	-	05:01	06:04	03:01	04:02
6KBPYU8AF	01:01	03:01	01:01:02G	-	-	-	-	-	02:01	05:01	03:01	04:02
6MYBPSV3E	03:01	04:04	01:01:02G	-	01:01:01G	-	-	-	02:01	03:02	01:01	04:02
6PBX7932E	03:01	04:04	01:01:02G	-	01:01:01G	-	-	-	02:01	03:02	01:01	20:01
6TYGSHNE8	07:01	12:01	02:02:01G	-	01:01:01G	-	-	-	02:01	03:01	02:01	04:01
6VTMPHA5B	07:01	15:03	-	-	01:01:01G	-	01:01:01G	-	02:01	06:02	01:01	02:01
6W8Z93NJB	01:03	11:01	02:02:01G	-	-	-	-	-	03:01	03:01	04:02	06:01
6Y7RHU9VB	03:01	15:01	01:01:02G	-	-	-	01:01:01G	-	02:01	06:02	01:01	04:01
6Z7WVAREG	11:01	13:02	02:02:01G	03:01:01G	-	-	-	-	03:01	06:04	03:01	04:02
6ZjNbSTcG	07:01	13:01	01:01:02G	-	01:01:01G	-	-	-	03:03	06:03	03:01	04:01
72JDMjNxc	01:01	04:01	-	-	01:01:01G	-	-	-	03:01	05:01	04:01	04:02
74TN5R3XJ	09:01	11:01	02:02:01G	-	01:01:01G	-	-	-	03:01	03:03	03:01	03:01
758BACRSW	04:01	13:02	03:01:01G	-	01:01:01G	-	-	-	03:02	06:04	01:01	02:01
76U8CJ9AF	12:02	13:02	03:01:01G	-	-	-	-	-	03:01	06:04	04:01	21:01
78AMVDQT6	07:01	09:01	-	-	01:01:01G	-	-	-	02:01	03:03	04:02	14:01
79BAHR58Z	13:02	16:01	03:01:01G	-	-	-	02:02:01G	-	05:02	06:04	04:02	04:02
79PGR3CQH	04:01	15:01	-	-	01:01:01G	-	01:01:01G	-	03:01	06:02	04:01	23:01
7BC9HPWQ6	04:01	15:01	-	-	01:01:01G	-	01:01:01G	-	03:01	06:02	04:01	13:01
7BFPX3KET	13:01	15:01	01:01:02G	-	-	-	01:01:01G	-	06:02	06:03	10:01	13:01
7CA4E56ZF	01:01	01:01	-	-	-	-	-	-	05:01	05:01	04:01	04:02
7EYW5QZCT	07:01	08:02	-	-	01:01:01G	-	-	-	02:01	03:01	04:02	04:02
7FSMEU8NA	03:01	14:04	02:02:01G	-	-	-	-	-	02:01	05:03	04:01	04:01
7K68ZG2YV	03:01	04:01	01:01:02G	-	01:01:01G	-	-	-	02:01	03:02	04:01	04:01
7KGXY8D6V	01:01	15:02	-	-	-	-	01:02:01G	-	05:01	06:01	04:01	10:01
7MCR894N6	01:01	08:02	-	-	-	-	-	-	04:02	05:01	04:01	04:01
7MCUXYSPZ	15:03	16:02	-	-	-	-	01:01:01G	02:02:01G	05:02	06:02	18:01	85:01
7NFA9ZEQP	13:02	15:01	03:01:01G	-	-	-	01:01:01G	-	06:02	06:04	02:01	04:01
7PFEJGN4A	01:01	11:01	02:02:01G	-	-	-	-	-	03:01	05:01	04:01	04:01
7PVSEUMH9	11:04	13:02	02:02:01G	03:01:01G	-	-	-	-	03:01	06:04	02:01	04:02
7R5YHA3NW	13:01	14:04	02:02:01G	-	-	-	-	-	05:03	06:03	04:02	17:01
7RVWXHJ2Q	04:04	07:01	-	-	01:01:01G	-	-	-	03:02	03:03	04:01	04:01
7TZPH6Q9S	13:02	14:01	02:02:01G	03:01:01G	-	-	-	-	05:03	06:04	04:01	17:01
7UDNZAT8Q	11:04	15:02	02:02:01G	-	-	-	01:02:01G	-	03:01	06:01	04:01	26:01
7V84BTYSF	07:01	12:02	03:01:01G	-	01:01:01G	-	-	-	03:01	03:03	10:01	21:01
7WZS3NJTU	04:07	13:01	02:02:01G	-	01:01:01G	-	-	-	03:01	06:02	02:01	19:01

7XK3WCP9N	04:05	13:12	02:02:01G	-	01:01:01G	-	-	-	03:01	04:01	05:01	14:01
7Y65NSBPT	13:03	15:01	01:01:02G	-	-	-	01:01:01G	-	03:01	06:02	04:01	17:01
7ZQ6FAPXH	15:01	15:01	-	-	-	-	01:01:01G	-	06:02	06:02	04:01	04:02
82AYNG3TH	07:01	07:01	-	-	01:01:01G	-	-	-	02:01	03:03	04:01	04:02
83BCG6X5R	03:02	11:01	01:01:02G	02:02:01G	-	-	-	-	04:02	06:11	01:01	18:01
85E3NH6JM	04:04	04:05	-	-	01:01:01G	-	-	-	03:02	03:02	04:02	13:01
86AN7SF43	15:01	15:01	-	-	-	-	01:01:01G	-	06:02	06:02	01:01	04:01
86VSGXNTH	01:01	07:01	-	-	01:01:01G	-	-	-	02:01	05:01	04:01	11:01
872VGYRDE	03:01	10:01	01:01:02G	-	-	-	-	-	02:01	05:01	04:01	04:02
89EA37HFY	04:04	15:01	-	-	01:01:01G	-	01:01:01G	-	03:02	06:02	04:01	06:01
8AZ6VMFNX	01:01	11:04	02:02:01G	-	-	-	-	-	03:01	05:01	04:01	04:02
8B5Q9RFPZ	01:01	03:01	01:01:02G	-	-	-	-	-	02:01	05:01	04:01	10:01
8BQ6Z9UKX	04:05	13:01	01:01:02G	-	01:01:01G	-	-	-	03:02	06:03	04:01	19:01
8BVDK2UYG	07:01	11:01	02:02:01G	-	01:01:01G	-	-	-	02:01	06:02	04:01	17:01
8DR7XM4QG	11:02	12:01	01:01:02G	02:02:01G	-	-	-	-	03:01	05:01	18:01	29:01
8EWJ7TVFA	03:02	15:01	01:01:02G	-	-	-	01:01:01G	-	04:02	06:33	02:01	03:01
8GWRKSAYM	03:01	14:01	01:01:02G	02:02:01G	-	-	-	-	02:01	05:03	05:01	09:01
8HAYN9XFQ	11:04	16:01	02:02:01G	-	-	-	02:02:01G	-	03:01	05:02	02:01	03:01
8HJV7CNFW	03:02	04:07	01:01:02G	-	01:01:01G	-	-	-	03:02	04:02	11:01	14:01
8MHJVPE67	04:01	13:02	03:01:01G	-	01:01:01G	-	-	-	03:01	06:04	04:01	04:02
8PKRCEV9Y	07:01	15:01	-	-	01:01:01G	-	01:01:01G	-	02:01	06:02	01:01	04:02
8V46KGPJX	11:01	14:01	02:02:01G	-	-	-	-	-	03:01	05:03	04:01	04:02
8YAWZKHB3	12:01	13:01	02:02:01G	-	-	-	-	-	03:01	06:03	04:01	04:02
8YtDhZQ4B	04:01	15:01	-	-	01:01:01G	-	01:01:01G	-	03:02	06:02	02:02	04:01
8Z9Y5VF2Q	03:01	13:02	01:01:02G	03:01:01G	-	-	-	-	02:01	06:09	04:01	04:02
8ZDX6MFNW	01:01	03:01	01:01:02G	-	-	-	-	-	02:01	05:01	04:01	13:01
8ZSBUA9F4	03:01	15:01	02:02:01G	-	-	-	01:01:01G	-	02:01	06:02	03:01	04:01
974PD2ABK	01:02	15:01	-	-	-	-	01:01:01G	-	05:01	06:02	04:01	04:01
9BEP25ZUX	04:04	08:01	-	-	01:01:01G	-	-	-	03:02	04:02	03:01	04:01
9BGNEDHKT	07:01	12:01	02:02:01G	-	01:01:01G	-	-	-	03:01	03:03	04:02	13:01
9BZEHXGWN	01:01	01:01	-	-	-	-	-	-	05:01	05:01	16:01	19:01
9CS465H2R	01:01	15:01	-	-	-	-	01:01:01G	-	05:01	06:02	04:02	23:01
9D5VQYA6J	04:01	07:01	-	-	01:01:01G	-	-	-	02:01	03:02	04:02	04:02
9D85X7JQ3	03:01	15:02	01:01:02G	-	-	-	01:02:01G	-	02:01	06:01	04:01	04:02
9dzNYDX8u	07:01	10:01	-	-	01:01:01G	-	-	-	02:01	05:01	04:01	10:01
9GU4ZDHQE	01:01	03:01	01:01:02G	-	-	-	-	-	02:01	05:01	01:01	02:01
9h3Gs2tKR	01:01	13:02	03:01:01G	-	-	-	-	-	05:01	06:09	04:02	05:01
9Hx5SfNse	01:01	07:01	-	-	01:01:01G	-	-	-	02:01	05:01	04:02	11:01
9J6NC4G2P	13:01	15:02	02:02:01G	-	-	-	01:01:01G	-	05:02	06:03	19:01	31:01
9JT8XVBHQ	03:01	15:01	02:02:01G	-	-	-	01:01:01G	-	02:01	06:02	01:01	17:01
9MGPThrZK	03:01	09:01	01:01:02G	-	01:01:01G	-	-	-	02:01	03:03	03:01	03:01
9N48H7SVQ	04:04	15:01	-	-	01:01:01G	-	01:01:01G	-	03:02	06:02	03:01	04:02
9NY7BSMGJ	01:01	04:07	-	-	01:01:01G	-	-	-	03:01	05:01	04:01	04:01
9Q7BD5F6N	03:01	07:01	02:02:01G	-	01:01:01G	-	-	-	02:01	02:01	04:02	17:01
9QD2VSWHG	01:01	04:08	-	-	01:01:01G	-	-	-	03:01	05:01	04:01	04:01
9QK7JFDGP	03:01	08:02	01:01:02G	-	-	-	-	-	02:01	04:02	03:01	04:01
9TKU85VQW	11:01	15:03	03:01:01G	-	-	-	01:01:01G	-	05:02	06:02	11:01	18:01
9U2T56EFG	01:01	01:01	-	-	-	-	-	-	05:01	05:01	02:01	04:02
9W8ATS3HM	01:01	15:03	-	-	-	-	01:01:01G	-	05:01	06:02	01:01	04:01
9XQFK6E3D	03:01	11:01	01:01:02G	02:02:01G	-	-	-	-	02:01	03:01	04:01	04:01
9Y4NUHM8B	03:01	08:01	01:01:02G	-	-	-	-	-	02:01	04:02	01:01	10:01
9YNVPSK2Z	01:01	13:01	01:01:02G	-	-	-	-	-	05:01	06:03	03:01	04:01
9YPEW28X4	08:01	13:01	01:01:02G	-	-	-	-	-	04:02	06:03	03:01	04:01
9Z73XKP5A	11:04	13:02	02:02:01G	03:01:01G	-	-	-	-	03:01	06:09	04:02	06:01
9zndEBxRK	04:07	15:01	-	-	01:01:01G	-	01:01:01G	-	03:01	06:02	04:01	04:01
A2HRGKE85	04:56	15:01	-	-	01:01:01G	-	01:01:01G	-	03:02	06:02	02:01	04:01
A32X5QSUE	07:01	12:02	03:01:01G	-	01:01:01G	-	-	-	02:01	05:02	13:01	13:01

A3WKDTNV4	07:01	16:01	-	-	01:01:01G	-	02:02:01G	-	02:01	05:02	04:01	04:01
A4HXMKY2C	04:07	15:01	-	-	01:01:01G	-	01:01:01G	-	03:02	06:02	04:02	10:01
A4XGE2V7J	03:01	04:04	01:01:02G	-	01:01:01G	-	-	-	02:01	03:02	01:01	03:01
A53YDNZS2	03:01	07:01	01:01:02G	-	01:01:01G	-	-	-	02:01	03:03	04:01	23:01
A67ZN3WR8	15:02	16:02	-	-	-	-	01:03	02:02:01G	05:02	05:02	02:02	05:01
A6QWDE5UX	03:02	10:01	01:01:02G	-	-	-	-	-	04:02	05:01	01:01	10:01
A925CXBTU	03:02	15:03	01:01:02G	-	-	-	01:01:01G	-	04:02	06:02	01:01	01:01
AE9CFPKZM	04:01	13:01	02:02:01G	-	01:01:01G	-	-	-	03:01	06:03	03:01	04:01
AEWQMB3DH	09:01	12:01	01:01:02G	-	01:01:01G	-	-	-	03:01	03:03	05:01	13:01
AGERCUJNX	01:01	15:03	-	-	-	-	01:01:01G	-	05:01	06:02	01:01	04:01
AGJETMCV3	07:01	16:01	-	-	01:01:01G	-	02:02:01G	-	02:01	05:02	02:01	04:02
AGMSUV5HT	09:01	11:01	02:02:01G	-	01:07:01	-	-	-	03:01	03:03	04:02	05:01
AM5U93XDJ	03:01	11:01	01:01:02G	02:02:01G	-	-	-	-	02:01	03:01	01:01	04:01
ANQHRP6JC	15:01	15:01	-	-	-	-	01:01:01G	-	06:02	06:02	04:01	04:01
ANR32YTX4	03:01	04:08	02:02:01G	-	01:01:01G	-	-	-	02:01	03:01	04:01	04:01
APCR5H4V3	04:01	04:05	-	-	01:01:01G	-	-	-	03:02	03:02	04:01	13:01
AQEHS54NF	15:03	15:03	-	-	-	-	01:01:01G	-	06:02	06:02	18:01	39:01
AQF3SV82R	09:01	11:01	02:02:01G	-	01:01:01G	-	-	-	03:03	06:02	04:01	18:01
AR62PZF4U	04:03	08:02	-	-	01:01:01G	-	-	-	03:02	04:02	04:02	17:01
AR7UZSQ9K	07:01	13:01	01:01:02G	-	01:01:01G	-	-	-	02:01	06:03	13:01	20:01
ARCMHYJGQ	07:01	13:01	01:01:02G	-	01:01:01G	-	-	-	02:01	06:03	03:01	11:01
AS94NMFED	15:01	16:01	-	-	-	-	01:01:01G	02:02:01G	05:02	06:02	04:01	04:01
AST3DHEX2	07:01	13:03	01:01:02G	-	01:01:01G	-	-	-	03:01	03:03	02:01	04:01
ATN7KR6BD	13:02	14:01	02:02:01G	03:01:01G	-	-	-	-	05:03	06:09	04:01	05:01
ATtXwe8ZB	07:01	15:01	-	-	01:01:01G	-	01:01:01G	-	02:01	06:02	02:01	11:01
AU4VQPRYS	03:02	13:02	01:01:02G	03:01:01G	-	-	-	-	04:02	05:01	01:01	04:02
AWUT2C543	07:01	13:02	03:01:01G	-	01:01:01G	-	-	-	03:03	06:04	01:01	04:01
AZSPKH2QN	07:01	11:01	02:02:01G	-	01:01:01G	-	-	-	02:01	03:01	02:01	04:01
B25KGZXSC	13:03	15:01	02:02:01G	-	-	-	01:01:01G	-	02:01	06:02	04:01	11:01
B3NM9ZYSK	04:01	04:04	-	-	01:01:01G	-	-	-	03:01	03:02	04:01	04:01
B3PHYFRKE	07:01	13:03	01:01:02G	-	01:01:01G	-	-	-	02:01	03:01	04:01	04:02
B43KTARJ2	08:04	13:02	03:01:01G	-	-	-	-	-	03:01	06:09	03:01	04:01
B4PR7QYWZ	01:03	13:02	03:01:01G	-	-	-	-	-	05:01	06:04	04:01	10:01
B8VWRUAG9	04:03	11:01	02:02:01G	-	01:01:01G	-	-	-	03:01	03:02	02:01	02:01
B9AV6QK7E	01:01	04:01	-	-	01:01:01G	-	-	-	03:02	05:01	04:01	04:01
B9DJ83K5T	04:03	12:02	03:01:01G	-	01:01:01G	-	-	-	03:01	03:02	02:02	05:01
B9HX8YK7N	08:04	11:01	02:02:01G	-	-	-	-	-	04:02	06:02	02:01	18:01
B9PY8ZRF5	08:03	15:01	-	-	-	-	01:01:01G	-	06:01	06:02	04:01	05:01
BCEYNDZTP	01:01	11:01	02:02:01G	-	-	-	-	-	03:01	05:01	04:01	04:01
BD3UQ95NE	01:01	01:01	-	-	-	-	-	-	05:01	05:01	04:01	04:02
BEM4G5N7A	04:01	07:01	-	-	01:01:01G	-	-	-	02:01	03:01	04:01	10:01
BH3NCAQUE	01:03	15:01	-	-	-	-	01:01:01G	-	05:01	06:02	02:01	04:01
BJ42RKCXY	04:07	04:07	-	-	01:01:01G	-	-	-	03:02	03:02	04:02	04:02
BJU9N7RD8	11:03	15:01	02:02:01G	-	-	-	01:01:01G	-	03:01	06:02	04:01	11:01
BKX7VC8MY	14:01	16:02	02:02:01G	-	-	-	02:02:01G	-	03:01	05:03	02:01	04:02
BQZDF5TEU	04:07	13:03	01:01:02G	-	01:01:01G	-	-	-	03:01	03:01	01:01	03:01
BRQ5KP23M	04:01	08:04	-	-	01:01:01G	-	-	-	03:01	03:01	01:01	04:01
BRZW7SNX2	04:01	15:01	-	-	01:01:01G	-	01:01:01G	-	03:01	06:02	04:01	04:01
BSC6UK5RN	13:02	15:01	03:01:01G	-	-	-	01:01:01G	-	06:02	06:09	03:01	04:01
BSDZVPAJU	01:01	15:01	-	-	-	-	01:01:01G	-	05:01	06:02	04:01	04:02
BSN5UYZK6	11:01	13:02	02:02:01G	03:01:01G	-	-	-	-	03:01	06:04	04:01	04:01
BSWYRQNJ2	03:01	11:01	01:01:02G	02:02:01G	-	-	-	-	02:01	03:01	04:01	04:01
BTGQCMD5N	03:01	15:01	02:02:01G	-	-	-	01:01:01G	-	02:01	03:01	02:01	05:01
BTGXAS95D	11:03	11:04	02:02:01G	-	-	-	-	-	03:01	03:01	04:01	11:01
BW8T26NP3	01:02	03:01	01:01:02G	-	-	-	-	-	02:01	05:01	02:01	04:01
BWFPQYH3U	01:01	03:01	01:01:02G	-	-	-	-	-	02:01	05:01	02:01	04:01
BYM4VA92R	01:03	13:01	01:01:02G	-	-	-	-	-	05:01	06:03	02:01	02:01

BYWGHM28T	04:04	11:02	02:02:01G	-	01:01:01G	-	-	-	03:01	03:02	02:01	14:01
BYXTVD6KR	01:01	03:01	02:02:01G	-	-	-	-	-	02:01	05:01	01:01	04:01
BZE9RA8NC	01:01	03:01	02:02:01G	-	-	-	-	-	02:01	05:01	04:01	15:01
BZUDWPXFQ	11:03	15:01	02:02:01G	-	-	-	01:01:01G	-	03:01	06:02	04:01	04:01
BZWG6QJT4	10:01	13:01	01:01:02G	-	-	-	-	-	05:01	06:03	03:01	11:01
C248AUNW7	01:03	04:07	-	-	01:01:01G	-	-	-	03:02	05:01	02:01	04:02
c2Xx6DtaK	04:04	07:01	-	-	01:01:01G	-	-	-	03:02	03:03	02:01	04:01
C3SYBKERG	01:01	13:02	03:01:01G	-	-	-	-	-	05:01	06:04	04:01	06:01
C4NDE2JYZ	01:01	01:01	-	-	-	-	-	-	05:01	05:01	02:01	04:01
C4YMNBE6A	04:04	04:05	-	-	01:01:01G	-	-	-	02:01	03:02	02:01	04:01
c5pQyuaDv	04:01	07:01	-	-	01:01:01G	-	-	-	03:02	03:03	03:01	04:01
C6A42MP7T	03:01	14:01	01:01:02G	02:02:01G	-	-	-	-	02:01	05:03	04:01	04:01
C6pYHfyds	13:02	15:01	03:01:01G	-	-	-	01:01:01G	-	05:01	05:01	04:01	63:01
CAT8SVPK3	04:03	13:01	01:01:02G	-	01:01:01G	-	-	-	03:04	06:03	02:01	02:01
CAUD4G32Z	03:01	15:01	02:02:01G	-	-	-	01:01:01G	-	02:01	06:02	02:01	04:01
CEGZRTM46	04:07	08:01	-	-	01:01:01G	-	-	-	03:02	04:02	03:01	04:02
CgqG4naW9	11:03	15:01	02:02:01G	-	-	-	01:01:01G	-	03:01	06:02	04:01	04:01
CJ4VTEN9H	12:01	13:03	01:01:02G	02:02:01G	-	-	-	-	02:01	05:01	01:01	18:01
CJKAVBHMG	11:01	11:01	02:02:01G	-	-	-	-	-	03:01	03:01	04:01	04:02
CJMAFUS6G	11:04	13:02	02:02:01G	03:01:01G	-	-	-	-	03:01	06:04	01:01	02:01
CJwvM5ZDX	11:04	15:01	02:02:01G	-	-	-	01:01:01G	-	03:01	06:02	03:01	05:01
CK789UGWN	01:01	07:01	-	-	01:01:01G	-	-	-	03:03	05:01	02:01	04:01
cku2Hhx3E	01:02	15:03	-	-	-	-	01:01:01G	-	05:01	06:02	02:01	03:01
CMFQKBZSG	03:01	15:01	01:01:02G	-	-	-	01:01:01G	-	02:01	06:02	04:01	04:01
CMR9W23PK	11:01	12:01	01:01:02G	02:02:01G	-	-	-	-	05:01	06:02	04:01	18:01
CPDKTFWR5	04:01	13:02	03:01:01G	-	01:01:01G	-	-	-	03:01	06:04	02:01	04:01
CQD3Z2FWJ	07:01	15:01	-	-	01:01:01G	-	01:01:01G	-	03:03	06:02	04:01	05:01
CRS28MWDV	01:01	01:01	-	-	-	-	-	-	05:01	05:01	02:02	04:01
CSMWRX89A	04:03	11:04	02:02:01G	-	01:01:01G	-	-	-	03:01	03:02	04:01	04:01
ctau4Fs2n	11:01	13:02	02:02:01G	03:01:01G	-	-	-	-	03:01	06:09	04:01	14:01
CTAWERBZG	13:02	14:01	02:02:01G	03:01:01G	-	-	-	-	05:03	06:09	04:01	05:01
CTKREB3PD	15:01	15:01	-	-	-	-	01:01:01G	-	06:02	06:02	04:01	04:01
CTVHDKNM9	07:01	11:01	02:02:01G	-	01:01:01G	-	-	-	02:01	03:01	01:01	04:01
CUKWPRA34	07:01	12:01	02:02:01G	-	01:01:01G	-	-	-	02:01	03:01	04:02	04:02
CVAR9347J	01:02	15:01	-	-	-	-	01:01:01G	-	05:01	06:02	04:01	04:01
CWG35N7JQ	08:02	15:03	-	-	-	-	01:01:01G	-	04:02	06:02	02:01	04:02
CZ679VGJX	04:01	04:07	-	-	01:01:01G	-	-	-	03:01	03:02	04:01	04:02
Cz7H8qnDY	07:01	13:02	03:01:01G	-	01:01:01G	-	-	-	02:01	06:04	04:01	17:01
CZNBK2F6M	03:01	15:01	01:01:02G	-	-	-	01:01:01G	-	02:01	06:02	04:01	04:01
D27HUJCEB	08:01	13:02	03:01:01G	-	-	-	-	-	04:02	06:04	04:01	04:01
D2HGMU56W	04:05	07:01	-	-	01:01:01G	-	-	-	02:01	02:01	03:01	04:01
D5A93B2WY	08:04	16:02	-	-	-	-	02:02:01G	-	03:01	05:02	02:01	85:01
D6rT3HcGx	04:01	08:04	-	-	01:01:01G	-	-	-	03:02	04:02	02:01	03:01
D84S3UXPQ	03:01	04:03	01:01:02G	-	01:01:01G	-	-	-	02:01	03:02	01:01	04:01
D8NWK2YQJ	03:01	11:01	01:01:02G	02:02:01G	-	-	-	-	02:01	03:01	04:01	04:01
D8YTQEKA4	03:01	09:01	01:01:02G	-	01:01:01G	-	-	-	02:01	03:03	01:01	03:01
D92ZSMWXT	03:01	09:01	01:01:02G	-	01:01:01G	-	-	-	02:01	02:01	01:01	04:01
DGbxlMH6O	07:01	10:01	-	-	01:01:01G	-	-	-	02:01	05:01	04:01	17:01
DGF5Y6Z7U	07:01	07:01	-	-	01:01:01G	-	-	-	02:01	02:01	01:01	02:01
DGVF3K6QZ	03:01	04:02	02:02:01G	-	01:01:01G	-	-	-	02:01	03:02	02:01	02:01
DK876QVYP	03:01	15:01	01:01:02G	-	-	-	01:01:01G	-	02:01	06:02	01:01	04:01
DKFYNE73S	04:01	13:01	02:02:01G	-	01:01:01G	-	-	-	03:01	06:09	03:01	04:02
DMBJQT52V	01:01	07:01	-	-	01:01:01G	-	-	-	02:01	05:01	04:01	04:02
DQARS3ZP8	11:01	13:03	01:01:02G	02:02:01G	-	-	-	-	03:01	06:02	01:01	02:01
DQBY32MKA	01:01	04:02	-	-	01:01:01G	-	-	-	03:02	05:01	04:01	04:01
DQCVFZ459	03:01	10:01	01:01:02G	-	-	-	-	-	02:01	05:01	03:01	04:01
DQHXJESTY	03:01	14:02	01:01:02G	-	-	-	-	-	02:01	03:01	01:01	04:02

DR5EVYJMC	11:01	13:03	01:01:02G	02:02:01G	-	-	-	-	03:01	03:01	02:01	17:01
DRKVU3H6C	04:01	04:01	-	-	01:01:01G	-	-	-	03:01	03:02	02:01	04:02
DS64TRJB5	08:01	15:01	-	-	-	-	01:01:01G	-	04:02	06:02	05:01	05:01
DUCRQ7KB2	04:04	11:01	02:02:01G	-	01:01:01G	-	-	-	03:01	03:02	02:01	03:01
DUJ8KYPVS	04:04	15:01	-	-	01:01:01G	-	01:01:01G	-	03:02	06:02	04:01	10:01
DUWHZV8PG	07:01	12:01	02:02:01G	-	01:01:01G	-	-	-	02:01	05:01	04:02	18:01
DV3PS48Q5	04:04	15:01	-	-	01:01:01G	-	01:01:01G	-	03:02	06:02	02:01	04:01
DV684PWGY	11:01	13:03	01:01:02G	02:02:01G	-	-	-	-	03:01	03:01	02:01	17:01
DXGkaJWyF	01:02	13:02	03:01:01G	-	-	-	-	-	05:01	06:09	05:01	05:01
E2c7nzysp	01:02	11:01	02:02:01G	-	-	-	-	-	03:01	05:01	03:01	04:02
E2RBZQFTG	01:01	13:01	01:01:02G	-	-	-	-	-	05:01	06:03	03:01	06:01
E3axnNczH	12:01	13:03	01:01:02G	-	-	-	-	-	03:01	03:01	04:02	04:02
E3F2YD58C	01:01	07:01	-	-	01:01:01G	-	-	-	03:03	05:01	03:01	04:01
E4UAS9VGY	04:04	07:01	-	-	01:01:01G	-	-	-	02:01	03:02	03:01	11:01
E4XBJP56S	07:01	15:01	-	-	01:01:01G	-	01:01:01G	-	02:01	06:01	03:01	04:01
E5GCA29S6	04:01	14:01	02:02:01G	-	01:01:01G	-	-	-	03:01	05:03	02:01	03:01
E64C8UPGW	01:01	13:02	03:01:01G	-	-	-	-	-	05:01	06:04	03:01	03:01
E65FVBA2U	01:01	03:01	01:01:02G	-	-	-	-	-	02:01	05:01	04:01	04:01
E7GQDH8UR	01:01	07:01	-	-	01:01:01G	-	-	-	02:01	05:01	04:01	11:01
E86FSM5GK	07:01	14:01	02:02:01G	-	01:01:01G	-	-	-	02:01	05:03	04:01	04:02
E9W67J42V	01:01	04:01	-	-	01:01:01G	-	-	-	03:01	05:01	04:01	04:01
EA3BHV46J	01:01	11:01	02:02:01G	-	-	-	-	-	03:01	05:01	04:01	04:01
EAzjNqySZ	11:04	13:02	02:02:01G	03:01:01G	-	-	-	-	03:01	06:04	03:01	17:01
EBC7YQR6S	07:01	07:01	-	-	01:01:01G	-	-	-	02:01	03:03	04:01	13:01
EBFC78J39	13:02	13:03	02:02:01G	03:01:01G	-	-	-	-	02:01	06:04	01:01	04:01
ECdP584uy	01:01	04:01	-	-	01:01:01G	-	-	-	03:01	05:01	04:01	04:02
ECnpskYMt	11:01	12:01	01:01:02G	02:02:01G	-	-	-	-	03:01	05:01	04:01	11:01
edqD6cFRm	01:03	04:04	-	-	01:01:01G	-	-	-	03:02	05:01	02:01	04:01
egKnUwXqR	01:02	03:01	01:01:02G	-	-	-	-	-	05:01	06:02	02:01	04:02
EGrMQ2zsW	07:01	13:01	01:01:02G	-	01:01:01G	-	-	-	03:03	06:03	02:01	13:01
ehTrXK9Vu	04:01	15:01	-	-	01:01:01G	-	01:01:01G	-	03:01	06:02	04:01	04:01
EHYPFWJVN	04:04	14:06	01:01:02G	-	01:01:01G	-	-	-	03:01	03:02	04:02	04:02
eHzBxTfwS	13:01	15:01	01:01:02G	-	-	-	01:01:01G	-	06:02	06:03	02:01	10:01
EJDXRTA83	08:02	14:06	01:01:02G	-	-	-	-	-	03:01	04:02	04:01	04:02
EK7VCAPFZ	11:01	13:01	01:01:02G	02:02:01G	-	-	-	-	03:01	06:03	04:01	04:01
EKP6F8SV4	11:01	12:02	03:01:01G	-	-	-	-	-	03:01	05:02	01:01	02:02
Em6UrcN78	01:01	13:01	02:02:01G	-	-	-	-	-	05:01	06:03	03:01	19:01
EMDYG3S4H	07:01	11:04	02:02:01G	-	01:01:01G	-	-	-	02:01	03:01	04:01	11:01
ENMQPCVTY	15:01	15:03	-	-	-	-	01:01:01G	-	06:02	06:02	03:01	18:01
ENSQ38RPX	01:01	13:01	01:01:02G	-	-	-	-	-	05:01	06:03	03:01	04:02
ENT2YXS7V	07:01	14:01	02:02:01G	-	01:01:01G	-	-	-	02:01	05:03	02:01	05:01
EP6S4Z7AD	04:04	04:05	-	-	01:01:01G	-	-	-	03:02	03:02	03:01	06:01
ESJFKZV9N	01:01	04:01	-	-	01:01:01G	-	-	-	03:02	05:01	04:01	04:01
ESZTGRBJ5	03:01	13:01	01:01:02G	02:02:01G	-	-	-	-	02:01	06:08	03:01	18:01
etB7Zdz3c	11:01	13:01	01:01:02G	02:02:01G	-	-	-	-	03:01	06:03	03:01	17:01
ETWU6R2ZC	13:01	15:01	02:02:01G	-	-	-	01:01:01G	-	06:02	06:03	04:01	04:01
EUZG35KQ4	08:03	15:01	-	-	-	-	01:01:01G	-	03:01	06:02	04:01	04:02
EVT9GZDQC	04:04	04:04	-	-	01:01:01G	-	-	-	03:02	03:02	04:01	06:01
EW3Q2UM9H	13:02	15:01	03:01:01G	-	-	-	01:01:01G	-	06:02	06:04	03:01	04:01
EWJNMUVZG	03:01	03:02	01:01:02G	02:02:01G	-	-	-	-	02:01	04:02	01:01	01:01
EX2P4FTJB	01:03	13:01	02:02:01G	-	-	-	-	-	05:01	06:03	02:01	04:01
EYGQ8RUXT	04:11	08:06	-	-	01:01:01G	-	-	-	03:02	06:02	03:01	04:02
EZTK6NRFW	04:02	13:01	02:02:01G	-	01:01:01G	-	-	-	03:02	06:03	04:01	04:01
F2JXTCVS3	07:01	12:01	01:01:02G	-	01:01:01G	-	-	-	02:01	05:01	01:01	18:01
F2M8WH6S3	14:06	15:01	01:01:02G	-	-	-	01:01:01G	-	03:01	06:02	04:02	05:01
F5ZSWAN9G	13:01	15:01	02:02:01G	-	-	-	01:01:01G	-	06:02	06:03	04:01	04:01
F6P5WYQUE	07:01	15:01	-	-	01:01:01G	-	01:01:01G	-	02:01	05:02	04:01	11:01

F7KYERMN5	03:01	13:01	01:01:02G	02:02:01G	-	-	-	-	02:01	06:03	01:01	04:01
FAT8K97QP	01:01	15:03	-	-	-	-	01:01:01G	-	05:01	05:03	04:01	04:02
FB5JRNM7X	03:01	13:05	02:02:01G	-	-	-	-	-	02:01	03:01	04:01	14:01
FDMCV72G3	08:02	11:01	02:02:01G	-	-	-	-	-	02:01	04:02	04:01	04:02
FDTNJPY9M	04:02	07:01	-	-	01:01:01G	-	-	-	02:01	03:02	02:01	05:01
FESBDPT9R	03:01	13:02	01:01:02G	03:01:01G	-	-	-	-	02:01	06:09	02:01	02:01
FETJAZ5V8	04:01	07:01	-	-	01:01:01G	-	-	-	02:01	03:02	04:01	04:02
FJ2B6ADRQ	04:01	11:04	02:02:01G	-	01:01:01G	-	-	-	03:01	03:02	04:01	04:02
FJY2Q3EXV	04:01	11:01	02:02:01G	-	01:01:01G	-	-	-	03:01	03:01	03:01	15:01
FK82C7MG5	03:01	07:01	01:01:02G	-	01:01:01G	-	-	-	02:01	02:01	04:01	23:01
FKYJ79CPR	11:01	15:01	02:02:01G	-	-	-	01:01:01G	-	03:01	06:02	03:01	04:01
fm5Xk3MC7	11:03	15:01	02:02:01G	-	-	-	01:01:01G	-	03:01	06:02	04:01	04:01
FMBYC73TU	04:07	08:02	-	-	01:01:01G	-	-	-	03:02	04:02	04:02	04:02
FP3BZQSDK	07:01	15:01	-	-	01:01:01G	-	01:01:01G	-	02:01	06:02	10:01	13:01
FP62WQJ9R	07:01	13:02	03:01:01G	-	01:01:01G	-	-	-	02:01	06:09	01:01	04:02
FPS7QB69J	01:01	15:01	-	-	-	-	01:01:01G	-	05:01	06:02	04:01	04:01
FQZ9y7c4W	07:01	15:01	-	-	01:01:01G	-	01:01:01G	-	02:01	06:02	04:01	11:01
FRKU9PNVZ	03:02	11:01	01:01:02G	02:02:01G	-	-	-	-	04:02	06:02	01:01	04:02
FT5CN4ZBP	04:01	13:02	03:01:01G	-	01:01:01G	-	-	-	03:01	06:04	04:01	04:02
FTUE6HZMY	04:01	10:01	-	-	01:01:01G	-	-	-	03:01	05:01	04:01	17:01
FU3BEPG2A	03:01	07:01	02:02:01G	-	01:01:01G	-	-	-	02:01	02:01	02:01	15:01
FUJvtah7X	04:01	07:01	-	-	01:01:01G	-	-	-	03:03	03:05	04:01	04:01
FVTMC3U4E	03:01	04:01	01:01:02G	-	01:01:01G	-	-	-	02:01	03:02	03:01	04:01
FWS32RTD7	15:01	15:01	-	-	-	-	01:01:01G	-	06:02	06:02	04:01	04:01
FX8HDACV3	14:01	15:01	02:02:01G	-	-	-	01:01:01G	-	05:03	06:02	02:01	03:01
FXRKNPA5Y	01:01	04:01	-	-	01:01:01G	-	-	-	03:02	05:01	04:02	23:01
g2HRmqnMV	08:04	13:01	01:01:02G	-	-	-	-	-	03:01	06:03	02:01	04:02
G4AMPXNCB	03:01	11:01	01:01:02G	02:02:01G	-	-	-	-	02:01	03:01	06:01	09:01
G57YXRQ32	07:01	11:01	02:02:01G	-	01:01:01G	-	-	-	02:01	03:03	04:01	04:02
G64K7UQ2R	04:01	04:11	-	-	01:01:01G	-	-	-	03:01	03:02	03:01	04:02
G7XA34EKC	07:01	14:01	02:02:01G	-	01:01:01G	-	-	-	02:01	05:03	02:01	04:01
G8HN7BFWA	13:01	15:01	01:01:02G	-	-	-	01:01:01G	-	06:02	06:03	03:01	04:01
G94HT7XV5	03:01	04:03	01:01:02G	-	01:01:01G	-	-	-	02:01	03:02	04:01	04:02
GAEMDXP8N	03:01	11:01	01:01:02G	02:02:01G	-	-	-	-	02:01	03:01	01:01	04:02
GBZH9YAVX	12:02	13:02	03:01:01G	-	-	-	-	-	03:01	05:01	01:01	05:01
GCYRQ7FU5	11:01	11:04	02:02:01G	-	-	-	-	-	03:01	03:01	04:01	10:01
GCZHQKXU8	01:01	14:05	02:02:01G	-	-	-	-	-	05:01	05:03	02:01	05:01
GD6NRS7WY	10:01	11:04	02:02:01G	-	-	-	-	-	03:01	05:01	04:01	04:02
GE9RQUNCJ	04:01	15:01	-	-	01:01:01G	-	01:01:01G	-	03:02	06:02	04:01	05:01
GEV9KUWY6	04:07	13:01	02:02:01G	-	01:01:01G	-	-	-	03:02	03:03	04:02	04:02
GF38ZE2QR	07:01	13:02	03:01:01G	-	01:01:01G	-	-	-	02:01	06:09	04:01	05:01
gGt5aPB7b	04:07	13:02	03:01:01G	-	01:01:01G	-	-	-	03:01	06:09	03:01	05:01
gGTR8xNYl	03:01	09:01	01:01:02G	-	01:01:01G	-	-	-	02:01	03:03	01:01	23:01
GH9KMFJQN	01:01	03:01	01:01:02G	-	-	-	-	-	02:01	05:01	04:01	11:01
GHCUVQZS7	07:01	07:01	-	-	01:01:01G	-	-	-	02:01	02:01	04:02	05:01
GJ5K9PNMA	01:01	11:01	02:02:01G	-	-	-	-	-	03:01	05:01	04:01	04:01
GKZTFV7S2	01:01	11:01	02:02:01G	-	-	-	-	-	03:01	05:01	04:01	11:01
GNTME9BVK	12:01	12:01	02:02:01G	-	-	-	-	-	03:01	05:01	04:01	04:01
gp2f83DPw	03:01	04:04	01:01:02G	-	01:01:01G	-	-	-	02:01	03:02	03:01	06:01
GpaXCtYBk	03:01	13:01	01:01:02G	02:02:01G	-	-	-	-	02:01	06:03	02:01	04:02
GQR39EVKJ	09:01	15:01	-	-	01:01:01G	-	01:01:01G	-	03:03	06:02	02:01	04:01
GS4DC3MRV	04:01	11:01	02:02:01G	-	01:01:01G	-	-	-	03:01	03:02	04:01	04:01
GSTPJ7WQA	04:07	15:01	-	-	01:01:01G	-	01:01:01G	-	03:02	06:02	04:01	04:02
GT6KCPS8R	04:01	04:01	-	-	01:01:01G	-	-	-	03:01	03:02	02:01	04:02
GUHB7DQKF	01:01	04:07	-	-	01:01:01G	-	-	-	03:02	05:01	03:01	10:01
GUNRZ9XYM	01:01	11:01	02:02:01G	-	-	-	-	-	03:01	05:01	04:02	05:01
gUsXCcWTQ	03:01	07:01	01:01:02G	-	01:01:01G	-	-	-	02:01	02:01	01:01	04:01

GV8RF4WH5	04:04	13:02	03:01:01G	-	01:01:01G	-	-	-	03:02	06:04	02:01	06:01
GZQ4VXDAM	04:01	15:01	-	-	01:01:01G	-	01:01:01G	-	03:02	06:02	04:01	16:01
H2XB5CPRG	07:01	11:04	02:02:01G	-	01:01:01G	-	-	-	02:01	03:01	04:01	04:02
H3AXC9YVB	01:03	04:04	-	-	01:01:01G	-	-	-	03:02	05:01	04:01	04:01
H3MCWFT8R	04:01	04:05	-	-	01:01:01G	-	-	-	03:01	03:02	02:01	04:01
H4JY5CMG8	07:01	13:01	02:02:01G	-	01:01:01G	-	-	-	03:03	03:03	01:01	04:01
H4ZEK2GBJ	04:04	04:07	-	-	01:01:01G	-	-	-	03:02	03:02	04:02	04:02
H65397KZV	03:01	10:01	01:01:02G	-	-	-	-	-	02:01	05:01	04:01	04:01
H7EGCTZYU	01:01	04:07	-	-	01:01:01G	-	-	-	03:01	05:01	03:01	04:02
H8RE76GSM	01:01	01:01	-	-	-	-	-	-	05:01	05:01	04:01	04:01
HAUGY8NJV	07:01	07:01	-	-	01:01:01G	-	-	-	03:03	03:03	04:01	13:01
HC275N9RW	04:05	15:01	-	-	01:01:01G	-	01:01:01G	-	02:01	06:02	04:01	05:01
HC5GU7PZM	13:02	16:02	03:01:01G	-	-	-	02:02:01G	-	05:02	06:09	02:01	17:01
HEFTNXAMB	09:01	13:01	02:02:01G	-	01:01:01G	-	-	-	03:03	06:03	02:01	02:01
HGA2KRMQ9	03:01	11:04	02:02:01G	-	-	-	-	-	02:01	03:01	02:01	04:02
HGQZW4NKV	03:01	13:01	01:01:02G	02:02:01G	-	-	-	-	02:01	06:03	10:01	13:01
HGSRDJYZP	11:04	15:02	02:02:01G	-	-	-	01:02:01G	-	03:01	06:01	04:01	26:01
HJ7KN6TSC	04:02	11:04	02:02:01G	-	01:01:01G	-	-	-	03:01	03:02	04:01	04:02
HJAMVRBE6	01:01	01:01	-	-	-	-	-	-	05:01	05:01	04:01	04:02
HJBSCN9UA	09:01	14:04	02:02:01G	-	01:01:01G	-	-	-	03:03	05:03	04:02	05:01
HJFWS7T5P	07:01	15:01	-	-	01:01:01G	-	01:01:01G	-	02:01	06:02	02:01	19:01
HK4EQDJS2	15:01	15:01	-	-	-	-	01:01:01G	-	05:02	06:02	02:01	04:01
HMB4TZN8X	04:04	15:01	-	-	01:01:01G	-	01:01:01G	-	03:02	06:02	02:01	04:01
hN9MJufBZ	04:01	04:07	-	-	01:01:01G	-	-	-	03:01	03:02	03:01	03:01
HP3ZNJVC5	04:01	11:01	02:02:01G	-	01:01:01G	-	-	-	03:01	03:02	04:01	04:01
HPAYDF43T	01:01	15:01	-	-	-	-	01:01:01G	-	05:01	06:02	04:01	04:01
HPCUF79XK	04:01	08:01	-	-	01:01:01G	-	-	-	03:02	04:02	04:01	04:01
HPRN3AV8K	04:04	07:01	-	-	01:01:01G	-	-	-	03:02	03:03	06:01	13:01
HQDYZ4TB9	13:01	13:01	02:02:01G	-	-	-	-	-	06:03	06:03	04:02	19:01
HRGSF2Z9U	03:01	07:01	02:02:01G	-	01:01:01G	-	-	-	02:01	02:01	04:01	04:01
HS6PK52TA	11:03	15:01	02:02:01G	-	-	-	01:01:01G	-	03:01	06:02	04:01	06:01
hSyzGJ698	11:01	15:01	02:02:01G	-	-	-	01:01:01G	-	03:01	06:02	04:01	04:01
HU8BEGX4P	04:04	07:01	-	-	01:01:01G	-	-	-	02:01	03:02	04:01	11:01
Hv2PcBdAF	03:01	07:01	01:01:02G	-	01:01:01G	-	-	-	02:01	02:01	02:01	04:01
HVGE8MJPA	03:02	11:11	01:01:02G	02:02:01G	-	-	-	-	03:01	04:02	01:01	04:02
HW5yZgE9C	04:04	13:01	02:02:01G	-	01:01:01G	-	-	-	03:02	06:03	04:02	06:01
HXP8S9YKB	11:04	11:04	02:02:01G	-	-	-	-	-	03:01	03:01	04:01	15:01
HXVUDEBT9	07:01	15:01	-	-	01:01:01G	-	01:01:01G	-	02:01	06:03	04:01	04:02
HXZSWMUDB	04:03	11:01	02:02:01G	-	01:01:01G	-	-	-	03:01	03:02	04:01	14:01
HY52KTRP7	04:04	13:02	03:01:01G	-	01:01:01G	-	-	-	03:02	06:04	02:01	05:01
HZBRKTC2W	04:11	14:01	02:02:01G	-	01:01:01G	-	-	-	03:02	05:03	02:01	04:02
HZqQra5bD	03:01	11:04	02:02:01G	-	-	-	-	-	02:01	03:01	02:02	03:01
J29K5FC4U	01:03	03:01	01:01:02G	-	-	-	-	-	02:01	05:01	02:02	04:02
j2BZCHKRa	12:02	12:02	03:01:01G	-	-	-	-	-	03:01	03:01	03:01	13:01
J2HPXGFBD	01:01	04:01	-	-	01:01:01G	-	-	-	03:02	05:01	03:01	10:01
J2RYPMN4U	07:01	07:01	-	-	01:01:01G	-	-	-	02:01	02:01	04:02	17:01
J4G2SDT63	07:01	16:01	-	-	01:01:01G	-	02:02:01G	-	02:01	05:02	04:02	11:01
J4N6BATR7	04:01	04:01	-	-	01:01:01G	-	-	-	03:02	03:02	04:01	04:02
J5C8R7MPX	03:02	04:01	01:01:02G	-	01:01:01G	-	-	-	03:01	04:02	01:01	04:01
J83TWX4ZF	03:01	15:01	01:01:02G	-	-	-	01:01:01G	-	02:01	06:02	01:01	04:01
J86ZTBR3A	04:04	13:01	01:01:02G	-	01:01:01G	-	-	-	03:02	06:03	04:02	04:02
J8M35AHCY	01:01	15:01	-	-	-	-	01:01:01G	-	05:01	06:02	04:01	10:01
J9XHNBCVK	01:01	13:02	03:01:01G	-	-	-	-	-	05:04	06:09	01:01	03:01
JBM2YCQPU	01:02	13:01	02:02:01G	-	-	-	-	-	05:01	06:03	04:01	06:01
JCTEXWKV2	07:01	11:04	02:02:01G	-	01:01:01G	-	-	-	03:01	03:03	13:01	13:01
JETDVB2X8	08:03	15:01	-	-	-	-	01:01:01G	-	03:01	06:02	02:01	10:01
JGKRCYZ5N	04:05	11:01	02:02:01G	-	01:01:01G	-	-	-	03:01	04:01	05:01	05:01

JGNBK6SYA	04:11	15:01	-	-	01:01:01G	-	01:01:01G	-	03:02	06:02	04:01	04:02
jGQnbh83v	04:04	13:01	01:01:02G	-	01:01:01G	-	-	-	03:02	06:03	04:01	04:01
JHX3ZRUP5	04:04	13:02	03:01:01G	-	01:01:01G	-	-	-	03:02	05:02	04:02	04:02
jMeWHNpSb	03:01	08:04	02:02:01G	-	-	-	-	-	02:01	03:01	03:01	11:01
JMWR2U534	01:01	07:01	-	-	01:01:01G	-	-	-	02:01	05:04	03:01	11:01
JNCKQ59V2	01:01	11:03	02:02:01G	-	-	-	-	-	03:01	05:01	04:02	04:02
JnwkMfT2e	04:01	04:07	-	-	01:01:01G	-	-	-	03:01	03:02	03:01	04:01
JQ5X9MGKE	04:05	13:02	03:01:01G	-	01:01:01G	-	-	-	03:02	06:04	01:01	02:01
JQDXNAKS8	03:01	12:02	02:02:01G	03:01:01G	-	-	-	-	02:01	03:01	04:01	05:01
JQHAreMa2	04:01	16:01	-	-	01:01:01G	-	02:02:01G	-	03:02	05:02	04:01	04:01
JQNETX38B	01:01	08:04	-	-	-	-	-	-	03:01	05:01	03:01	04:02
JQR8MF7DC	07:01	12:02	02:02:01G	-	01:01:01G	-	-	-	02:01	06:02	02:01	04:02
JQW63NRBX	13:01	15:01	02:02:01G	-	-	-	01:01:01G	-	06:02	06:03	04:01	04:02
JSiLRogVs	01:01	04:04	-	-	01:01:01G	-	-	-	03:02	05:01	02:01	04:02
JTXVP6DES	12:01	15:01	02:02:01G	-	-	-	01:01:01G	-	03:01	06:02	04:01	14:01
JVESH846A	11:01	13:02	02:02:01G	03:01:01G	-	-	-	-	03:01	06:04	04:02	17:01
jVTMDARve	01:02	03:01	02:02:01G	-	-	-	-	-	02:01	05:01	02:01	02:01
JWN2GCD8H	04:07	15:01	-	-	01:01:01G	-	01:01:01G	-	03:01	06:02	04:01	04:01
JXAC6ZHMT	09:01	13:01	01:01:02G	-	01:01:01G	-	-	-	03:03	06:03	03:01	04:02
JXMCVYWSZ	07:01	11:01	02:02:01G	-	01:01:01G	-	-	-	02:01	03:01	02:01	04:02
JXYQD8U7T	01:01	15:01	-	-	-	-	01:01:01G	-	05:01	06:02	01:01	03:01
JZE8RCT2F	08:04	13:02	03:01:01G	-	-	-	-	-	04:02	05:01	01:01	02:01
K38HD6A9Z	07:01	15:01	-	-	01:01:01G	-	01:01:01G	-	03:03	06:02	02:01	04:01
K4PUN6ETG	08:04	16:01	-	-	-	-	02:02:01G	-	03:01	05:02	03:01	04:01
K4U7R5JVX	01:02	07:01	-	-	01:01:01G	-	-	-	02:01	05:01	04:02	05:01
k4WFsZg8a	11:01	15:01	02:02:01G	-	-	-	01:01:01G	-	03:01	06:02	04:01	04:01
K5HGR76AW	01:01	07:01	-	-	01:01:01G	-	-	-	02:01	05:01	03:01	11:01
K5ZBMF2UA	11:01	13:03	01:01:02G	02:02:01G	-	-	-	-	03:01	03:01	04:01	04:01
K8ZUPG59S	03:01	13:02	01:01:02G	03:01:01G	-	-	-	-	02:01	06:04	01:01	04:01
KACPYGBN8	04:01	11:01	02:02:01G	-	01:01:01G	-	-	-	03:01	03:02	02:01	04:01
KAUT4H3RN	07:01	09:01	-	-	01:01:01G	-	-	-	02:01	03:02	02:01	04:02
KBQCWA93F	04:04	13:02	03:01:01G	-	01:01:01G	-	-	-	03:02	06:04	02:01	04:01
KC6Y3Z7XR	01:01	04:01	-	-	01:01:01G	-	-	-	03:02	05:01	04:01	13:01
KCQJZ6PRD	15:01	15:01	-	-	-	-	01:01:01G	-	06:02	06:02	04:01	04:02
KDAMEHFQP	01:01	04:01	-	-	01:01:01G	-	-	-	03:01	05:01	03:01	04:01
KDB5M897C	07:01	13:02	03:01:01G	-	01:01:01G	-	-	-	02:01	06:04	01:01	01:01
KDH84TZJN	01:02	04:01	-	-	01:01:01G	-	-	-	03:02	05:01	01:01	04:01
KE4XQD7SZ	01:03	13:02	03:01:01G	-	-	-	-	-	05:01	06:04	02:01	04:01
KFQNDRHGM	04:04	15:01	-	-	01:01:01G	-	01:01:01G	-	03:02	06:02	04:01	04:01
KG2DCW5NB	03:01	11:01	02:02:01G	-	-	-	-	-	02:01	06:02	02:01	03:01
kgaJ5qHRj	07:01	07:01	-	-	01:01:01G	-	-	-	02:01	03:03	02:01	02:01
KH5T3QXSF	04:07	13:02	03:01:01G	-	01:01:01G	-	-	-	03:01	06:04	03:01	04:02
KH7GA8J2U	15:01	15:03	-	-	-	-	01:01:01G	-	06:02	06:02	04:01	18:01
KHGY3CXAU	04:01	07:01	-	-	01:01:01G	-	-	-	02:01	03:01	04:01	04:02
KHX4DAW9V	03:01	14:01	02:02:01G	-	-	-	-	-	02:01	05:03	01:01	01:01
KJ43XS2TD	04:01	07:01	-	-	01:01:01G	-	-	-	03:01	03:03	04:01	04:01
KJFPD7C2T	13:02	14:02	01:01:02G	03:01:01G	-	-	-	-	03:01	06:04	04:01	04:02
KjGckxTtd	04:01	08:01	-	-	01:01:01G	-	-	-	03:02	04:02	04:01	04:01
KJypHj64a	01:01	07:01	-	-	01:01:01G	-	-	-	02:01	05:01	04:01	04:02
kKpsfu6Ej	01:03	15:01	-	-	-	-	01:01:01G	-	05:01	06:02	02:01	03:01
KMHCQ2S47	03:01	04:01	01:01:02G	-	01:01:01G	-	-	-	02:01	03:01	02:01	04:01
KNQ26PU87	04:01	13:01	01:01:02G	-	01:01:01G	-	-	-	03:01	06:03	03:01	04:01
KQ5JNRSAF	01:01	15:01	-	-	-	-	01:01:01G	-	05:01	06:02	04:01	06:01
KQMP5WXNY	03:01	10:01	02:02:01G	-	-	-	-	-	02:01	05:01	01:01	13:01
KR42CNG75	04:04	07:01	-	-	01:01:01G	-	-	-	02:01	03:02	04:01	09:01
KU2BFVPMA	11:04	13:01	01:01:02G	02:02:01G	-	-	-	-	03:01	06:03	02:01	04:01
KVDPZMUEQ	07:01	07:01	-	-	01:01:01G	-	-	-	02:01	03:03	04:01	11:01

KY9ZCEAN6	04:02	14:01	02:02:01G	-	01:01:01G	-	-	-	03:02	05:03	02:01	03:01
KYW79VUAQ	15:01	16:01	-	-	-	-	01:01:01G	02:02:01G	05:02	06:02	04:01	04:01
KZU5ECW9M	07:01	15:01	-	-	01:01:01G	-	01:01:01G	-	02:01	06:02	05:01	11:01
KZUA9X7F2	08:02	13:02	03:01:01G	-	-	-	-	-	03:02	05:01	04:02	17:01
M3B874VPN	07:01	15:01	-	-	01:01:01G	-	01:01:01G	-	02:01	06:01	04:01	28:01
M3D2HJNTW	01:02	13:01	02:02:01G	-	-	-	-	-	03:03	05:01	01:01	01:01
m3ejIsx1U	04:01	12:01	02:02:01G	-	01:01:01G	-	-	-	03:01	03:01	13:01	17:01
M3RTN98PE	11:01	11:04	02:02:01G	-	-	-	-	-	03:01	03:01	04:02	13:01
M4KZ9CHNR	01:01	07:01	-	-	01:01:01G	-	-	-	02:01	05:01	04:01	11:01
m5G9SFd23	11:04	13:02	02:02:01G	03:01:01G	-	-	-	-	03:01	06:04	02:01	03:01
M6FHDAZ8B	11:01	15:01	02:02:01G	-	-	-	01:01:01G	-	03:01	06:02	04:01	04:01
M78C4R3YG	04:04	04:07	-	-	01:01:01G	-	-	-	03:02	03:02	04:02	04:02
M925AXGVS	04:03	08:02	-	-	01:01:01G	-	-	-	03:02	04:02	03:01	04:02
MA4H37DXS	04:11	15:03	-	-	01:01:01G	-	01:01:01G	-	03:02	06:02	02:01	27:01
MBQSF2E8J	04:07	07:01	-	-	01:01:01G	-	-	-	02:01	03:01	04:01	04:02
MBUVSQEKZ	07:01	07:01	-	-	01:01:01G	-	-	-	02:01	02:01	04:01	04:01
MBWDUVPG6	07:01	13:02	03:01:01G	-	01:01:01G	-	-	-	02:01	06:04	03:01	11:01
MC4QTZHE6	08:02	13:05	02:02:01G	-	-	-	-	-	03:01	04:02	04:01	04:01
MCWB5Y8D3	04:01	08:03	-	-	01:01:01G	-	-	-	03:02	06:01	04:02	05:01
MEeoTUZ89	04:04	14:01	02:01:01G	-	01:01:01G	-	-	-	03:02	05:03	04:01	15:01
MGXBEQJDA	03:02	13:01	01:01:02G	-	-	-	-	-	04:02	06:03	01:01	04:02
MHEGC2NY5	04:01	04:01	-	-	01:01:01G	-	-	-	03:01	03:01	04:01	04:01
MJEHTQ4SY	01:01	04:04	-	-	01:01:01G	-	-	-	03:02	05:01	06:01	14:01
MKWQ9YJAB	01:01	12:01	01:01:02G	-	-	-	-	-	03:01	05:01	02:01	05:01
MkZOw5TXo	11:04	14:01	02:02:01G	-	-	-	-	-	03:01	05:03	04:01	04:02
MNUH7ZCFP	08:01	13:02	03:01:01G	-	-	-	-	-	04:02	06:09	02:01	04:01
MNZSV3UQP	03:01	04:03	01:01:02G	-	01:01:01G	-	-	-	02:01	03:02	01:01	04:01
mpVnE4tg5	01:03	13:02	03:01:01G	-	-	-	-	-	05:01	06:04	04:01	04:01
MQA4YCGRK	13:01	15:01	01:01:02G	-	-	-	01:01:01G	-	06:02	06:03	02:01	04:01
MRQ37GYP4	01:01	07:01	-	-	01:01:01G	-	-	-	02:01	05:01	04:01	11:01
MSJ5RZ9WK	03:01	04:01	01:01:02G	-	01:01:01G	-	-	-	02:01	03:02	01:01	04:01
MSJT3R42Y	15:01	15:02	-	-	-	-	01:01:01G	01:02:01G	06:01	06:02	04:01	09:01
MSRJPUW3Q	01:02	15:02	-	-	-	-	01:01:01G	-	05:01	06:01	04:01	04:01
MVDKRA86G	01:01	04:01	-	-	01:01:01G	-	-	-	03:02	05:01	02:01	04:02
MW26HBDJP	03:01	12:01	01:01:02G	02:02:01G	-	-	-	-	02:01	03:01	01:01	04:02
MW2BHDAX8	08:01	09:01	-	-	01:01:01G	-	-	-	02:01	04:02	04:01	13:01
MX3VRCPDF	03:02	13:01	01:01:02G	02:02:01G	-	-	-	-	04:02	06:04	01:01	04:01
MXFQZY42R	01:01	15:01	-	-	-	-	01:01:01G	-	05:01	06:02	03:01	04:01
MybeG9Ta3	01:02	03:01	01:01:02G	-	-	-	-	-	02:01	05:01	04:01	17:01
MYQHF547U	01:01	03:01	01:01:02G	-	-	-	-	-	02:01	05:01	03:01	09:01
MZ3R76K8H	07:01	11:04	02:02:01G	-	01:01:01G	-	-	-	02:01	03:01	01:01	04:02
N2RSPEHU8	01:01	01:01	-	-	-	-	-	-	05:01	05:01	03:01	04:02
N327F4XDY	04:01	16:02	-	-	01:01:01G	-	02:02:01G	-	03:01	03:01	04:01	04:02
N3MQXGA6P	13:05	15:03	02:02:01G	-	-	-	01:01:01G	-	03:01	06:02	02:01	04:02
N465C8FM2	01:01	07:01	-	-	01:01:01G	-	-	-	02:01	05:01	04:01	04:02
N4HP8SZYT	13:01	13:03	01:01:02G	-	-	-	-	-	03:01	06:03	11:01	14:01
N68B5QE4C	10:01	11:04	02:02:01G	-	-	-	-	-	03:01	05:01	04:01	04:02
N6BPgGTVb	13:01	15:01	01:01:02G	-	-	-	01:01:01G	-	06:02	06:03	04:01	04:01
N78FMHUW9	03:02	15:03	01:01:02G	-	-	-	01:01:01G	-	04:02	06:02	01:01	18:01
N84Q2XAPM	04:01	04:04	-	-	01:01:01G	-	-	-	03:02	03:02	04:01	04:01
N8XCMVETA	04:01	11:01	02:02:01G	-	01:01:01G	-	-	-	03:01	03:02	04:01	04:02
NASJ2X74P	04:01	07:01	-	-	01:01:01G	-	-	-	02:01	03:02	04:01	14:01
NCTFHMD3U	07:01	11:01	02:02:01G	-	01:01:01G	-	-	-	02:01	06:02	01:01	04:02
NCX9M5PV6	03:01	03:01	01:01:02G	-	-	-	-	-	02:01	02:01	01:01	01:01
NDH67FY8T	04:01	07:01	-	-	01:01:01G	-	-	-	02:01	03:02	04:01	04:01
NDT2BMVZW	11:01	13:02	02:02:01G	03:01:01G	-	-	-	-	03:01	06:04	03:01	14:01
NEFRM32YD	07:01	15:01	-	-	01:01:01G	-	01:01:01G	-	02:01	06:02	04:01	17:01

NEHWSCZMT	01:01	04:05	-	-	01:01:01G	-	-	-	02:01	05:01	03:01	04:01
NESW5TM2J	03:01	13:03	01:01:02G	-	-	-	-	-	02:01	03:01	03:01	04:02
NF6VDXE94	07:01	13:01	01:01:02G	-	01:01:01G	-	-	-	02:01	06:03	02:01	11:01
NHGT8F947	01:01	13:01	01:01:02G	-	-	-	-	-	05:01	06:03	02:01	04:01
NHSDT3ZW9	01:01	13:02	03:01:01G	-	-	-	-	-	05:01	06:09	03:01	04:01
NJ5QF9S3T	01:02	12:01	02:02:01G	-	-	-	-	-	03:01	05:01	04:01	04:02
NJVMC4Y6G	07:01	07:01	-	-	01:01:01G	-	-	-	02:01	03:03	02:01	15:01
NK7EAC9ak	07:01	10:01	-	-	01:01:01G	-	-	-	03:03	05:01	04:02	05:01
NM3ZHABQG	01:01	07:01	-	-	01:01:01G	-	-	-	02:01	05:01	04:01	04:02
NM6HZDB97	11:01	13:01	01:01:02G	02:02:01G	-	-	-	-	03:01	06:03	02:02	04:01
NP4QZTSVX	01:02	15:01	-	-	-	-	01:01:01G	-	05:01	06:02	04:01	04:01
NPU6T7BY5	03:01	04:01	01:01:02G	-	01:01:01G	-	-	-	02:01	03:02	02:01	02:01
NQRTW8Z6A	10:01	15:02	-	-	-	-	01:02:01G	-	05:01	06:01	03:01	04:01
NQYJ97PWG	13:02	15:01	03:01:01G	-	-	-	01:01:01G	-	06:02	06:09	04:01	04:01
NR6MFWUTS	04:04	08:01	-	-	01:01:01G	-	-	-	03:02	04:02	03:01	04:01
NRKWV27TH	01:01	14:01	02:02:01G	-	-	-	-	-	05:01	05:03	02:01	14:01
nsmpzEPJe	13:02	13:03	01:01:02G	03:01:01G	-	-	-	-	03:01	06:04	03:01	13:01
NSUQFY7HE	01:02	07:01	-	-	01:01:01G	-	-	-	03:03	05:01	04:01	13:01
NSV45C2WM	08:01	15:01	-	-	-	-	01:01:01G	-	04:02	06:02	03:01	04:01
NT2RU6XSF	07:01	13:02	03:01:01G	-	01:01:01G	-	-	-	02:01	06:04	04:02	11:01
NU8AJRPM5	01:01	04:01	-	-	01:01:01G	-	-	-	03:01	05:01	03:01	04:01
NWQ2HKP6B	04:01	13:02	03:01:01G	-	01:01:01G	-	-	-	03:01	06:04	02:01	03:01
NwRMGbJDr	03:01	11:01	01:01:02G	02:02:01G	-	-	-	-	02:01	03:01	02:01	04:01
NXP42SCVD	13:01	15:01	02:02:01G	-	-	-	01:01:01G	-	06:02	06:03	04:02	05:01
NYFMUXZG6	01:02	13:02	03:01:01G	-	-	-	-	-	05:01	05:01	02:01	11:01
P268BZEGA	03:01	15:01	01:01:02G	-	-	-	01:01:01G	-	02:01	06:02	01:01	04:01
P5CXAYS6G	03:01	14:01	01:01:02G	02:01:01G	-	-	-	-	02:01	05:03	01:01	04:01
P64YZ3GHQ	04:05	15:01	-	-	01:01:01G	-	01:01:01G	-	03:02	06:02	04:01	15:01
P72SHUW9Y	14:02	15:01	01:01:02G	-	-	-	01:01:01G	-	03:02	06:02	04:01	05:01
P7UWQ35SE	11:01	15:01	02:02:01G	-	-	-	01:01:01G	-	03:01	06:02	04:01	04:01
P89TZWXNS	01:03	07:01	-	-	01:01:01G	-	-	-	02:01	05:01	02:01	04:01
PAZBWYGJK	03:01	14:01	01:01:02G	02:02:01G	-	-	-	-	02:01	05:03	02:01	03:01
PDN58EKQV	13:01	15:01	02:02:01G	-	-	-	01:01:01G	-	06:02	06:03	04:01	10:01
PED85M9BF	03:01	11:01	01:01:02G	02:02:01G	-	-	-	-	02:01	03:01	04:01	04:01
PfherNtbn	03:01	13:02	01:01:02G	03:01:01G	-	-	-	-	02:01	06:04	03:01	04:01
PFNMUJYS8	01:01	09:01	-	-	01:01:01G	-	-	-	03:03	05:01	04:01	04:02
PH7CNM8KF	04:01	04:01	-	-	01:01:01G	-	-	-	03:02	03:02	03:01	04:01
PJ8X6NHTR	11:01	13:03	01:01:02G	02:02:01G	-	-	-	-	03:01	06:02	04:02	17:01
PM8DRY9EJ	07:01	13:01	01:01:02G	-	01:01:01G	-	-	-	03:03	06:03	04:01	04:02
PMQXR7UDY	11:04	13:01	01:01:02G	02:02:01G	-	-	-	-	03:01	06:03	04:01	09:01
PMYASJWE2	03:02	15:03	01:01:02G	-	-	-	01:01:01G	-	04:02	06:02	01:01	18:01
PNKE3RTSQ	04:01	07:01	-	-	01:01:01G	-	-	-	02:01	03:01	01:01	02:01
pqUHQMwfm	04:07	14:06	01:01:02G	-	01:01:01G	-	-	-	03:01	03:02	04:02	04:02
PSA68HFMU	01:01	15:01	-	-	-	-	01:01:01G	-	05:01	06:02	04:01	23:01
PsdqxHtCm	01:01	13:02	03:01:01G	-	-	-	-	-	05:01	06:04	02:01	02:01
PTESX598H	03:01	13:02	01:01:02G	03:01:01G	-	-	-	-	02:01	06:04	03:01	04:01
PU36WF4M8	01:01	07:01	-	-	01:01:01G	-	-	-	02:01	05:01	04:01	11:01
PVX3M6U4G	04:01	07:01	-	-	01:01:01G	-	-	-	03:01	03:03	04:01	13:01
PZG675JAM	01:01	07:01	-	-	01:01:01G	-	-	-	02:01	05:01	03:01	04:02
Q2NYHPW68	07:01	15:01	-	-	01:01:01G	-	01:01:01G	-	02:01	06:02	03:01	04:01
Q3RB4PTCV	03:01	15:02	02:02:01G	-	-	-	01:02:01G	-	02:01	06:01	02:02	04:02
Q3VYR24CS	03:02	11:01	01:01:02G	02:02:01G	-	-	-	-	03:01	04:02	01:01	03:01
Q547WRHZ3	12:01	15:01	01:01:02G	-	-	-	01:01:01G	-	03:01	06:02	02:01	05:01
Q598RFWYE	04:03	04:03	-	-	01:01:01G	-	-	-	03:02	03:02	04:01	04:01
Q5HAY8D3M	03:01	07:01	02:02:01G	-	01:01:01G	-	-	-	02:01	02:01	17:01	17:01
Q68ZDGVCH	04:01	13:01	01:01:02G	-	01:01:01G	-	-	-	03:02	06:03	04:01	04:01
Q6EXVSDHU	07:01	11:01	02:02:01G	-	01:01:01G	-	-	-	03:01	03:03	04:01	04:01

Q9DMNR8UK	04:05	15:01	-	-	01:01:01G	-	01:01:01G	-	03:02	06:02	04:01	04:01
QAFG8JSXD	04:01	13:01	01:01:02G	-	01:01:01G	-	-	-	03:01	06:03	02:01	04:01
QAG5E6CNB	03:01	13:01	01:01:02G	02:02:01G	-	-	-	-	02:01	06:03	09:01	19:01
QAMEW7JVP	04:01	11:01	02:02:01G	-	01:01:01G	-	-	-	03:01	03:01	01:01	04:01
QBA9M3T7D	08:02	15:02	-	-	-	-	01:02:01G	-	04:02	06:01	03:01	04:01
QG8UYJC5W	07:01	15:01	-	-	01:01:01G	-	01:01:01G	-	02:01	06:02	04:01	04:02
QGJAXHFZS	03:04	13:03	01:01:02G	-	-	-	-	-	02:01	03:01	04:01	04:01
QJDTAVUNK	03:02	08:04	01:01:02G	-	-	-	-	-	03:01	04:02	01:01	17:01
QKFWCNZD2	01:01	13:02	03:01:01G	-	-	-	-	-	05:01	06:04	04:01	04:01
QKHX5UMWP	01:02	03:01	01:01:02G	-	-	-	-	-	02:01	05:01	01:01	04:01
QKZ4T7FSX	07:01	15:01	-	-	01:01:01G	-	01:01:01G	-	02:01	06:02	03:01	04:01
QMVA7NP5X	14:04	14:04	02:02:01G	-	-	-	-	-	05:03	05:03	02:01	915:0
QnWeSYabF	04:01	12:01	02:02:01G	-	01:01:01G	-	-	-	03:01	03:02	04:01	14:01
QpzeaT9bf	11:04	13:01	01:01:02G	02:02:01G	-	-	-	-	03:01	06:03	03:01	04:01
QRMUKZEX9	11:01	13:02	02:02:01G	03:01:01G	-	-	-	-	03:01	06:04	04:01	18:01
QRPE8KGBD	08:01	11:01	02:02:01G	-	-	-	-	-	03:01	04:02	02:01	04:01
QSAX9MCEJ	03:01	09:01	01:01:02G	-	01:01:01G	-	-	-	02:01	03:03	01:01	05:01
QVK6RFTE7	04:04	04:04	-	-	01:01:01G	-	-	-	03:02	03:02	06:01	06:01
QWEXFB2JC	15:01	15:01	-	-	-	-	01:01:01G	-	06:02	06:02	04:01	04:01
QY3NSCR89	04:02	15:01	-	-	01:01:01G	-	01:01:01G	-	03:02	06:02	02:01	04:01
R36AQC8TZ	07:01	15:01	-	-	01:01:01G	-	01:01:01G	-	02:01	06:02	10:01	39:01
R4UQC958E	04:01	15:01	-	-	01:01:01G	-	01:01:01G	-	03:02	06:02	04:01	04:02
R4VQ2kmyJ	07:01	13:01	03:01:01G	-	01:01:01G	-	-	-	02:01	05:01	01:01	03:01
R53E9TJ7D	13:01	15:01	02:02:01G	-	-	-	01:01:01G	-	06:02	06:03	04:01	04:02
R5JAPXTNE	11:02	15:03	02:02:01G	-	-	-	01:01:01G	-	03:01	06:02	01:01	18:01
R6GKX2UBW	04:05	11:01	02:02:01G	-	01:01:01G	-	-	-	03:01	03:02	03:01	13:01
R8TNQHUPA	07:01	13:01	02:02:01G	-	01:01:01G	-	-	-	02:01	06:03	04:01	04:01
R8YXHFETA	01:02	13:05	02:02:01G	-	-	-	-	-	03:01	05:01	04:01	04:02
r9ZfTAnxd	01:01	10:01	-	-	-	-	-	-	05:01	05:01	02:01	05:01
RA9SK7Q4V	01:01	04:04	-	-	01:01:01G	-	-	-	03:02	05:01	04:01	06:01
RADGVHSCK	12:01	13:01	01:01:02G	02:02:01G	-	-	-	-	03:01	06:03	05:01	19:01
RF3KE7YAP	13:02	15:01	03:01:01G	-	-	-	01:01:01G	-	06:02	06:04	03:01	04:02
RFE7XMZ2C	07:01	15:01	-	-	01:01:01G	-	01:01:01G	-	03:03	05:01	03:01	04:01
RHM5K4SU6	04:01	13:02	03:01:01G	-	01:01:01G	-	-	-	03:02	06:09	03:01	05:01
RhVntgxYP	01:01	12:01	02:02:01G	-	-	-	-	-	03:01	05:01	01:01	02:01
RM78F5PSA	07:01	13:02	03:01:01G	-	01:01:01G	-	-	-	02:01	05:01	01:01	03:01
rMgVEH4dF	01:03	13:02	03:01:01G	-	-	-	-	-	05:01	06:04	02:01	04:01
RMY74CQAU	01:01	04:01	-	-	01:01:01G	-	-	-	03:01	05:01	04:01	04:01
RNQ5KADFS	08:06	09:01	-	-	01:01:01G	-	-	-	03:01	03:03	01:01	13:01
RNZAJ93GS	15:01	16:02	-	-	-	-	01:01:01G	-	05:02	06:02	05:01	05:01
RQ8M3BSZA	04:02	11:04	02:02:01G	-	01:01:01G	-	-	-	03:01	03:02	02:01	02:01
RQSJ863YA	13:02	13:03	01:01:02G	03:01:01G	-	-	-	-	03:01	06:09	04:01	05:01
RS9PUY586	04:01	15:01	-	-	01:01:01G	-	01:01:01G	-	03:01	06:02	04:01	04:01
RUKFNEH98	03:01	07:01	01:01:02G	-	01:01:01G	-	-	-	02:01	02:01	04:02	11:01
RUNZW8HSX	01:01	13:01	02:02:01G	-	-	-	-	-	05:01	06:03	04:01	04:02
RUVGYTXBD	03:01	13:04	01:01:02G	02:02:01G	-	-	-	-	02:01	03:01	01:01	17:01
RWN25394X	04:07	16:01	-	-	01:01:01G	-	02:02:01G	-	03:02	05:02	01:01	04:01
RYM7HS458	11:01	15:01	02:02:01G	-	-	-	01:01:01G	-	03:01	06:02	02:01	04:01
RZQPMTJYA	07:01	13:01	01:01:02G	-	01:01:01G	-	-	-	02:01	06:03	04:01	20:01
S2Q4XMRAP	04:10	09:01	-	-	01:01:01G	-	-	-	03:02	03:03	04:01	14:01
S6CDMHWXP	03:01	03:01	01:01:02G	02:02:01G	-	-	-	-	02:01	02:01	02:01	03:01
S6YG8CUF4	04:01	13:01	02:02:01G	-	01:01:01G	-	-	-	03:01	06:03	04:01	04:01
S8K6MJWVC	12:01	13:02	02:02:01G	03:01:01G	-	-	-	-	03:01	06:04	02:01	04:01
S9JBY34HP	04:01	07:01	-	-	01:01:01G	-	-	-	02:01	03:02	03:01	04:01
SA62V4MYD	03:01	15:01	02:02:01G	-	-	-	01:01:01G	-	02:01	06:02	04:01	05:01
SATB73R4Z	08:04	11:02	02:02:01G	-	-	-	-	-	03:01	03:01	03:01	29:01
SB4GEWTJ8	11:01	11:02	02:02:01G	-	-	-	-	-	03:01	06:02	03:01	04:01

SBTPDMVEJ	03:01	11:04	01:01:02G	02:02:01G	-	-	-	-	02:01	03:01	04:01	04:01
SDW78ZFU3	13:02	16:01	03:01:01G	-	-	-	02:02:01G	-	05:02	06:09	05:01	05:01
SFH5Y6RBK	13:01	15:01	01:01:02G	-	-	-	01:01:01G	-	06:02	06:03	04:01	04:01
SFJCN627X	08:01	15:01	-	-	-	-	01:01:01G	-	04:02	06:02	02:01	03:01
SG9YP8AKD	13:03	15:01	01:01:02G	-	-	-	01:01:01G	-	03:01	06:02	02:01	04:01
SGT4C8NWY	04:04	11:01	02:02:01G	-	01:01:01G	-	-	-	03:01	03:02	04:01	19:01
shVgfDbcm	08:01	15:01	-	-	-	-	01:01:01G	-	04:02	06:02	03:01	04:01
SJ2P95HVX	12:01	15:01	02:02:01G	-	-	-	01:01:01G	-	03:01	06:02	04:01	13:01
SM7KU6C39	11:01	13:03	01:01:02G	02:02:01G	-	-	-	-	03:01	03:01	04:01	18:01
sMBRhwm4D	15:01	15:01	-	-	-	-	01:01:01G	-	06:02	06:02	04:01	04:01
SMN4JWGQT	07:01	09:01	-	-	01:01:01G	-	-	-	02:01	03:03	11:01	14:01
sMTm6nEKd	14:04	15:01	02:02:01G	-	-	-	01:01:01G	-	05:03	06:02	04:01	04:01
SQ2T4X8U7	04:01	11:04	02:02:01G	-	01:01:01G	-	-	-	03:01	03:02	02:01	04:01
SQUJPM2Z5	08:04	13:02	03:01:01G	-	-	-	-	-	03:01	05:01	01:01	02:01
STMRDG5BC	15:01	15:01	-	-	-	-	01:01:01G	-	06:02	06:02	04:01	04:01
SU4WZF9MJ	09:01	14:05	02:02:01G	-	01:01:01G	-	-	-	03:03	05:03	05:01	38:01
SVUK7JPEM	08:01	15:01	-	-	-	-	01:01:01G	-	04:02	06:02	04:01	04:01
SWR2ETV7U	15:01	15:01	-	-	-	-	01:01:01G	-	05:02	06:02	04:01	06:01
SX8WKTYR4	01:01	13:03	01:01:02G	-	-	-	-	-	03:01	05:01	03:01	14:01
sxNDBwaAK	04:01	13:01	02:02:01G	-	01:01:01G	-	-	-	03:01	06:03	04:01	06:01
SYyQHk3sg	01:02	15:03	-	-	-	-	01:01:01G	-	05:01	06:02	01:01	04:01
SZ36CFVAQ	13:04	14:01	02:02:01G	-	-	-	-	-	03:01	05:02	01:01	17:01
T72CAUKP9	04:01	15:01	-	-	01:01:01G	-	01:01:01G	-	03:01	06:02	03:01	04:01
T7P3JQ2GR	11:01	11:03	02:02:01G	-	-	-	-	-	03:01	03:01	04:01	04:01
TAGQWE52B	08:04	14:01	02:02:01G	-	-	-	-	-	03:01	05:03	04:01	17:01
TB4SU2QJG	01:01	11:04	02:02:01G	-	-	-	-	-	03:01	05:01	02:01	14:01
TDG9MC5HK	03:02	11:01	01:01:02G	02:02:01G	-	-	-	-	04:02	06:02	02:01	85:01
TE3BVX9D8	07:01	12:01	02:02:01G	-	01:01:01G	-	-	-	02:01	03:01	04:01	05:01
TeCBDWFv5	03:01	04:01	01:01:02G	-	01:01:01G	-	-	-	02:01	03:02	02:01	02:01
teExZ3hCn	04:01	07:01	-	-	01:01:01G	-	-	-	02:01	03:01	04:01	06:01
TEZU3G7YV	01:01	15:01	-	-	-	-	01:01:01G	-	05:01	06:02	04:01	04:02
TGE7VP9CA	07:01	11:02	02:02:01G	-	01:01:01G	-	-	-	02:01	03:01	04:02	17:01
TGJP5HNS8	07:01	13:01	01:01:02G	-	01:01:01G	-	-	-	03:03	06:03	02:01	02:01
THRCUPY28	07:01	08:01	-	-	01:01:01G	-	-	-	02:01	04:02	04:01	17:01
TP4KXGCUR	01:01	07:01	-	-	01:01:01G	-	-	-	03:03	05:01	04:01	13:01
TrDAeYHJx	04:01	15:01	-	-	01:01:01G	-	01:01:01G	-	03:01	06:02	04:01	04:01
TUDEBNZCQ	07:01	13:02	03:01:01G	-	01:01:01G	-	-	-	03:03	06:04	03:01	16:01
TUNG3W4EM	10:01	13:01	01:01:02G	-	-	-	-	-	05:01	06:03	02:01	02:01
TVGJKZQU5	04:07	13:02	03:01:01G	-	01:01:01G	-	-	-	03:01	06:04	02:01	03:01
TVNSYX369	13:01	15:01	02:02:01G	-	-	-	01:01:01G	-	06:02	06:03	02:01	04:02
TVSZUWB3C	08:04	11:01	02:02:01G	-	-	-	-	-	03:01	06:02	01:01	04:02
TW8Q7ACP3	13:02	15:01	03:01:01G	-	-	-	01:01:01G	-	06:02	06:04	03:01	14:01
TYMSV7DZW	13:02	15:01	03:01:01G	-	-	-	01:01:01G	-	06:02	06:04	03:01	17:01
TZ8PGHF9A	07:01	16:01	-	-	01:01:01G	-	02:02:01G	-	02:01	05:02	04:01	04:01
U2JR74GXH	09:01	15:02	-	-	01:01:01G	-	01:02:01G	-	03:03	05:01	02:01	03:01
U34QDBGN8	01:01	13:03	01:01:02G	-	-	-	-	-	03:01	05:01	03:01	03:01
U48FAKEW5	03:04	07:01	01:01:02G	-	01:01:01G	-	-	-	02:01	02:01	02:01	16:01
U4YZCH7G2	03:01	12:01	01:01:02G	02:02:01G	-	-	-	-	02:01	03:01	01:01	04:02
U52DXET4P	11:01	13:01	02:02:01G	-	-	-	-	-	03:01	06:03	04:01	04:01
U5cuWtBVd	11:01	11:04	02:02:01G	-	-	-	-	-	03:01	03:01	02:01	02:01
U5YJX2BF7	04:01	11:01	02:02:01G	-	01:01:01G	-	-	-	03:01	03:02	04:01	14:01
U64TB8YV3	04:03	08:01	-	-	01:01:01G	-	-	-	03:02	04:02	04:01	04:02
U7NHX8K9J	04:07	08:02	-	-	01:01:01G	-	-	-	03:02	04:02	04:02	04:02
U94BNKQG8	11:04	15:01	02:02:01G	-	-	-	01:01:01G	-	03:01	06:02	04:01	04:02
U9GPWFZTB	01:01	04:07	-	-	01:01:01G	-	-	-	03:02	05:01	04:02	04:02
UB2QZXHPR	14:01	15:01	02:02:01G	-	-	-	01:01:01G	-	05:03	06:02	02:01	04:01
UBV5DFJ2R	04:01	13:03	01:01:02G	-	01:01:01G	-	-	-	03:01	03:01	02:01	02:01

uFpBfySTA	03:01	04:02	02:02:01G	-	01:01:01G	-	-	-	02:01	03:02	02:01	03:01
UG6SD52WZ	07:01	15:01	-	-	01:01:01G	-	01:01:01G	-	02:01	06:02	02:01	17:01
UG8EV7MT6	03:01	04:05	01:01:02G	-	01:01:01G	-	-	-	02:01	03:02	01:01	04:01
UHPCDV8RT	08:01	11:01	02:02:01G	-	-	-	-	-	03:01	04:02	04:01	04:01
UKDJGR92X	04:07	15:01	-	-	01:01:01G	-	01:01:01G	-	03:02	06:02	04:01	04:01
UMC6DXSG3	01:01	04:03	-	-	01:01:01G	-	-	-	03:02	05:01	04:01	09:01
UNC9EKJY4	04:07	14:06	01:01:02G	-	01:01:01G	-	-	-	03:01	03:02	04:02	04:02
UNTD2BHX9	08:02	11:04	02:02:01G	-	-	-	-	-	04:02	06:03	02:01	04:02
up65tBWyH	01:01	01:03	-	-	-	-	-	-	05:01	05:01	02:01	04:01
UPVQN2CKT	04:01	14:01	02:02:01G	-	01:01:01G	-	-	-	03:02	05:03	02:01	04:01
UQE567BTJ	07:01	13:02	03:01:01G	-	01:01:01G	-	-	-	03:03	06:04	04:01	04:01
URFTMW6QK	04:11	11:02	02:02:01G	-	01:01:01G	-	-	-	03:01	03:02	04:02	17:01
US62P9ZDR	10:01	13:01	02:02:01G	-	-	-	-	-	05:01	06:03	03:01	10:01
US73ANTXB	03:01	09:01	02:02:01G	-	01:01:01G	-	-	-	02:01	03:03	03:01	04:02
USN6RTJ8F	03:02	11:01	01:01:02G	02:02:01G	-	-	-	-	04:02	06:11	01:01	18:01
UTbaCgEmx	13:02	15:01	03:01:01G	-	-	-	01:01:01G	-	06:02	06:04	04:01	04:01
uymfkXe4w	03:01	15:01	01:01:02G	-	-	-	01:01:01G	-	02:01	06:02	01:01	04:01
V243BPWHA	09:01	15:02	-	-	01:01:01G	-	01:01:01G	-	03:03	05:02	04:01	05:01
V5H9K4WGE	07:01	07:01	-	-	01:01:01G	-	-	-	02:01	02:01	04:01	11:01
V7D58ACWM	09:01	15:03	-	-	01:01:01G	-	01:01:01G	-	02:01	06:02	18:01	18:01
V8Y5rZGbz	03:01	03:01	01:01:02G	-	-	-	-	-	02:01	02:01	01:01	04:01
V9G36AKNQ	03:01	09:01	01:01:02G	-	01:01:01G	-	-	-	02:01	03:03	01:01	04:01
V9HJGEYPD	03:01	03:01	01:01:02G	-	-	-	-	-	02:01	02:01	01:01	13:01
VA3MP459D	03:01	15:01	01:01:02G	-	-	-	01:01:01G	-	02:01	06:02	04:01	04:01
VA7KHWRGY	07:01	14:01	02:02:01G	-	01:01:01G	-	-	-	02:01	05:03	02:01	03:01
VB5X87KCW	13:02	13:04	02:02:01G	03:01:01G	-	-	-	-	03:01	06:04	04:01	17:01
vBcAdHfPm	03:01	10:01	01:01:02G	-	-	-	-	-	02:01	05:01	02:01	04:01
VBCP3Q7A5	04:03	07:01	-	-	01:01:01G	-	-	-	02:01	03:02	04:02	11:01
VGPFBSW94	11:01	11:04	02:02:01G	-	-	-	-	-	03:01	03:01	02:01	04:01
VHBQ52Y8U	14:01	16:02	02:01:01G	-	-	-	02:02:01G	-	03:01	05:03	02:01	04:01
VKTGH4EQF	11:01	13:01	02:02:01G	02:10	-	-	-	-	03:01	06:03	04:01	19:01
VMFA9KUQD	11:01	13:02	02:02:01G	03:01:01G	-	-	-	-	06:09	06:09	01:01	85:01
VMKQPUT25	01:02	13:02	03:01:01G	-	-	-	-	-	05:01	06:04	04:01	14:01
VMTU6R8AN	01:02	11:04	02:02:01G	-	-	-	-	-	03:01	05:01	04:01	04:01
VN76JDRQ9	15:01	15:01	-	-	-	-	01:01:01G	-	06:02	06:02	04:01	04:01
VNEQJ36WR	03:01	11:04	01:01:02G	02:02:01G	-	-	-	-	02:01	03:01	01:01	04:02
VNFK8PSX3	04:04	15:01	-	-	01:01:01G	-	01:01:01G	-	03:02	06:02	04:01	06:01
VPHD3G894	04:08	15:02	-	-	01:01:01G	-	01:02:01G	-	03:01	06:01	04:01	04:01
VQ3UGDZ4A	13:02	15:01	03:01:01G	-	-	-	01:01:01G	-	06:02	06:04	03:01	04:01
VQHME2W7C	07:01	07:01	-	-	01:01:01G	-	-	-	02:01	02:01	11:01	17:01
VRAB8XQNS	04:07	04:08	-	-	01:01:01G	-	-	-	03:01	03:01	03:01	04:01
VRZQAPC3F	07:01	15:01	-	-	01:01:01G	-	01:01:01G	-	03:03	06:02	02:01	04:01
VSCQ27M8N	13:02	15:01	03:01:01G	-	-	-	01:01:01G	-	06:02	06:04	04:01	09:01
VTFM6BSDX	14:01	15:03	02:02:01G	-	-	-	01:01:01G	-	05:03	06:02	02:01	04:01
VTGP5MEC3	04:01	09:01	-	-	01:01:01G	-	-	-	03:02	03:03	04:01	10:01
VU8DKT7ZG	15:01	15:01	-	-	-	-	01:01:01G	-	06:02	06:02	04:01	04:02
VUR5S9NBP	01:01	04:04	-	-	01:01:01G	-	-	-	03:02	05:01	04:01	04:01
VUWGAPYXE	01:01	04:01	-	-	01:01:01G	-	-	-	03:01	05:01	02:01	04:01
vWds8yEhn	04:04	11:01	02:02:01G	-	01:01:01G	-	-	-	03:01	03:02	04:01	06:01
VWR62NX8J	04:01	08:01	-	-	01:01:01G	-	-	-	03:01	04:02	02:01	03:01
VXCRM23YK	11:04	15:01	02:02:01G	-	-	-	01:01:01G	-	03:01	06:02	02:01	04:02
W23SJRBPV	01:02	11:03	02:02:01G	-	-	-	-	-	03:01	05:01	03:01	04:01
W2FPEHBRU	04:01	13:02	03:01:01G	-	01:01:01G	-	-	-	03:02	06:04	04:01	04:01
W34CVDR6Z	15:02	15:02	-	-	-	-	01:02:01G	-	05:01	06:01	13:01	13:01
W3NSCEFR8	01:01	13:03	01:01:02G	-	-	-	-	-	03:01	05:04	04:02	04:02
W57BTMGZN	07:01	12:01	02:02:01G	-	01:01:01G	-	-	-	02:01	05:01	03:01	11:01
W5KPAQXNT	04:04	13:04	02:02:01G	-	01:01:01G	-	-	-	03:01	03:02	02:01	17:01

W6DM3BES8	04:01	13:01	02:02:01G	-	01:01:01G	-	-	-	03:02	06:03	04:01	10:01
W7NC5DPGY	04:03	12:02	03:01:01G	-	01:01:01G	-	-	-	03:01	03:02	03:01	05:01
W7TDYFMKQ	08:03	15:02	-	-	-	-	01:01:01G	-	05:02	06:01	01:01	05:01
W8HKSEJDQ	07:01	15:01	-	-	01:01:01G	-	01:01:01G	-	03:03	06:02	04:01	04:01
W8XMCA7HS	08:04	16:01	-	-	-	-	02:02:01G	-	03:01	05:02	02:01	11:01
WAX34T2KV	03:01	11:04	01:01:02G	02:02:01G	-	-	-	-	02:01	03:01	01:01	04:01
WAXCDM9KN	12:01	15:01	02:02:01G	-	-	-	01:01:01G	-	03:01	06:02	04:01	04:01
wAXkvUtKf	11:01	13:02	02:02:01G	03:01:01G	-	-	-	-	03:01	06:04	03:01	04:02
WCnKmpPdq	04:04	08:04	-	-	01:01:01G	-	-	-	03:02	04:02	02:01	06:01
WFQ5D8CKT	04:04	07:01	-	-	01:01:01G	-	-	-	03:02	03:03	04:01	04:02
WGYRA4276	01:03	03:01	01:01:02G	-	-	-	-	-	02:01	03:01	04:01	04:01
WH3YQM8ZP	04:01	13:02	03:01:01G	-	01:01:01G	-	-	-	03:02	06:09	03:01	03:01
WJ36QRN8P	01:03	12:01	02:02:01G	-	-	-	-	-	03:01	05:01	04:02	14:01
WJPSGVA58	07:01	15:02	-	-	01:01:01G	-	01:02:01G	-	02:01	06:01	03:01	14:01
WP54UQS7X	04:04	13:02	03:01:01G	-	01:01:01G	-	-	-	03:02	06:04	03:01	06:01
WPBE48KX5	09:01	11:01	02:02:01G	-	01:01:01G	-	-	-	03:01	03:03	03:01	04:02
WpH6h4M7T	13:02	14:04	02:02:01G	03:01:01G	-	-	-	-	05:03	06:04	04:01	04:01
WQ5BY4T9S	15:01	16:01	-	-	-	-	01:01:01G	02:02:01G	05:02	06:02	02:01	04:01
WQVENRSP3	01:01	11:04	02:02:01G	-	-	-	-	-	03:01	05:01	04:01	04:02
WRY5P6F3N	03:02	04:04	01:01:02G	-	01:01:01G	-	-	-	03:02	04:02	01:01	02:01
wSNt2rCVG	03:01	12:01	01:01:02G	02:02:01G	-	-	-	-	02:01	03:01	04:02	04:02
WT3KQNF67	04:04	15:03	-	-	01:01:01G	-	01:01:01G	-	03:02	06:02	06:01	13:01
WVCXRK7J4	01:02	04:04	-	-	01:01:01G	-	-	-	03:02	05:01	01:01	03:01
WX4S269EF	04:07	15:02	-	-	01:01:01G	-	01:02:01G	-	03:02	06:01	02:02	04:02
WX9FQSNV6	07:01	13:04	02:02:01G	-	01:01:01G	-	-	-	02:01	03:01	03:01	17:01
WXN4DMU9B	03:01	14:02	01:01:02G	02:02:01G	-	-	-	-	02:01	03:01	02:01	04:02
WXNVY8FUG	04:10	08:02	-	-	01:01:01G	-	-	-	03:02	04:02	03:01	04:02
WY62H7KRZ	04:05	13:03	02:02:01G	-	01:01:01G	-	-	-	02:01	02:01	04:02	17:01
WYGEM9T3B	11:01	13:31	02:02:01G	03:01:01G	-	-	-	-	05:02	06:02	02:01	18:01
WYRS2JQ5E	03:01	11:04	01:01:02G	02:02:01G	-	-	-	-	02:01	03:01	01:01	03:01
WZAJ34GFU	04:02	13:01	01:01:02G	-	01:01:01G	-	-	-	03:02	06:03	04:01	19:01
WZTM23J7S	10:01	11:01	02:02:01G	-	-	-	-	-	03:01	05:01	04:02	17:01
X2DJE4RYN	11:01	15:03	02:02:01G	-	-	-	01:01:01G	-	06:02	06:02	02:01	17:01
X2MVCQDJE	04:05	09:01	-	-	01:01:01G	-	-	-	03:02	03:03	02:01	17:01
x3Yjp7rkV	11:04	13:01	02:02:01G	03:01:01G	-	-	-	-	05:01	05:02	03:01	17:01
X8D9HPCZ2	11:01	15:01	02:02:01G	-	-	-	01:01:01G	-	03:01	06:02	04:01	23:01
X9YEJB3GW	11:01	11:03	02:02:01G	-	-	-	-	-	03:01	03:01	03:01	04:01
XBWJ9C4DS	03:01	15:01	01:01:02G	-	-	-	01:01:01G	-	02:01	06:02	04:01	04:01
XCSWBQEM4	09:01	16:01	-	-	01:01:01G	-	02:02:01G	-	03:03	05:02	04:01	04:01
XDJMGNQ3E	04:01	07:01	-	-	01:01:01G	-	-	-	03:02	03:03	04:01	04:02
XEPGJS28Y	11:02	11:02	02:02:01G	-	-	-	-	-	03:01	03:01	01:01	04:02
XF5C4HN2B	01:01	15:02	-	-	-	-	01:02:01G	-	05:01	06:01	02:01	04:02
XFN3ZGPC2	07:01	11:04	02:02:01G	-	01:01:01G	-	-	-	02:01	03:01	10:01	13:01
XG2CY4ZP3	10:01	12:01	02:02:01G	-	-	-	-	-	03:01	05:01	02:01	18:01
XGQY2R69J	01:01	15:01	-	-	-	-	01:01:01G	-	05:01	06:02	04:01	04:02
XGU734FK6	12:01	13:01	02:02:01G	-	-	-	-	-	03:01	06:03	02:01	04:02
XHBGQ7FNR	07:01	11:01	02:02:01G	-	01:01:01G	-	-	-	02:01	03:01	02:01	05:01
XJ493SRQK	09:01	12:01	01:01:02G	-	01:01:01G	-	-	-	03:01	03:03	05:01	05:01
XJPZWK7TM	04:01	12:01	02:02:01G	-	01:01:01G	-	-	-	03:01	03:01	04:01	04:01
XM3Y8FDZS	04:01	04:38	-	-	01:01:01G	-	-	-	03:01	03:01	04:01	04:01
xMtHcwZbe	07:01	13:01	02:02:01G	-	01:01:01G	-	-	-	02:01	06:03	03:01	11:01
XPQZ7UM9G	13:01	15:01	02:02:01G	-	-	-	01:01:01G	-	06:02	06:03	04:01	04:01
XPSV8CZBJ	04:07	13:01	01:01:02G	-	01:01:01G	-	-	-	03:02	06:03	02:01	04:02
XPTC6Y2NA	12:01	13:01	01:01:02G	02:02:01G	-	-	-	-	03:01	06:03	03:01	04:01
XTGJZQCBE	03:01	13:04	01:01:02G	02:02:01G	-	-	-	-	02:01	03:01	04:01	13:01
XV54AR726	01:01	08:01	-	-	-	-	-	-	04:02	05:01	04:01	06:01
XVSZ8KNTJ	03:01	07:01	01:01:02G	-	01:01:01G	-	-	-	02:01	02:01	04:01	17:01

XVYJ7AMB9	01:01	13:02	03:01:01G	-	-	-	-	-	05:01	06:04	02:01	09:01
XWHS28D4Z	03:01	15:01	02:02:01G	-	-	-	01:01:01G	-	02:01	06:02	02:02	04:01
xWzke2K6c	04:07	12:01	02:02:01G	-	01:01:01G	-	-	-	03:01	03:01	04:02	04:02
xXkP3aDWz	04:07	08:03	-	-	01:01:01G	-	-	-	03:01	03:02	04:01	04:02
XY8SKQHJD	04:01	07:01	-	-	01:01:01G	-	-	-	02:01	03:02	04:01	05:01
XYF4GDM7B	15:01	15:01	-	-	-	-	01:01:01G	-	06:02	06:02	04:01	04:02
XYZR87KJV	04:07	07:01	-	-	01:01:01G	-	-	-	02:01	03:02	14:01	17:01
XZPKUDRN8	07:01	13:02	03:01:01G	-	01:01:01G	-	-	-	02:01	06:04	04:02	17:01
XZRYB38QC	04:04	07:01	-	-	01:01:01G	-	-	-	02:01	03:02	04:01	11:01
Y2BG37KHQ	15:01	15:01	-	-	-	-	01:01:01G	-	06:02	06:02	05:01	17:01
Y2XDU65Q4	04:02	04:03	-	-	01:01:01G	-	-	-	03:02	03:02	04:01	04:01
Y32URSWKZ	01:01	16:01	-	-	-	-	02:02:01G	-	05:01	05:02	04:02	10:01
Y39BXFKHS	04:01	11:01	02:02:01G	-	01:01:01G	-	-	-	03:01	03:02	02:01	15:01
Y3AMDQ6GX	04:04	11:04	02:02:01G	-	01:01:01G	-	-	-	03:01	03:02	02:01	06:01
Y3PUBE9XA	11:01	13:01	02:02:01G	-	-	-	-	-	06:02	06:03	02:01	14:01
y495UcZzK	07:01	12:01	02:02:01G	-	01:01:01G	-	-	-	02:01	05:01	01:01	02:01
Y5FMUKE8D	01:01	13:01	02:02:01G	-	-	-	-	-	05:01	06:03	03:01	19:01
Y68SG9QBE	07:01	11:01	02:02:01G	-	01:01:01G	-	-	-	02:01	03:01	04:01	04:02
Y6VN35BKA	03:01	04:07	01:01:02G	-	01:01:01G	-	-	-	02:01	03:02	04:02	355:0
Y7CW23GDT	04:04	04:07	-	-	01:01:01G	-	-	-	03:02	03:02	04:02	04:02
Y8QBZSFH7	01:01	15:01	-	-	-	-	01:01:01G	-	05:01	06:02	03:01	04:01
YADRU4B2F	13:03	15:01	01:01:02G	-	-	-	01:01:01G	-	03:01	06:02	02:01	04:01
YAUF4SXKM	04:11	13:02	03:01:01G	-	01:01:01G	-	-	-	04:02	06:04	04:01	04:02
YBJFQH7Z6	07:01	11:01	02:02:01G	-	01:01:01G	-	-	-	02:01	03:01	03:01	04:01
YBMUSN5VW	07:01	15:03	-	-	01:01:01G	-	01:01:01G	-	02:01	06:02	02:01	04:02
YC42XJSG9	08:02	13:01	03:01:01G	-	-	-	-	-	04:02	05:01	03:01	04:02
YCqyAZMbQ	07:01	15:01	-	-	01:01:01G	-	01:01:01G	-	03:03	06:02	04:01	13:01
YDFR6PMJB	01:01	10:01	-	-	-	-	-	-	05:01	05:01	01:01	03:01
YDTGPSAMV	01:01	08:01	-	-	-	-	-	-	04:02	05:01	03:01	06:01
YEVFT69AD	01:01	07:01	-	-	01:01:01G	-	-	-	02:01	05:01	03:01	04:02
YF8AUJ3EV	04:04	13:02	03:01:01G	-	01:01:01G	-	-	-	03:02	06:04	03:01	04:01
YFA7K2DNQ	01:01	11:04	02:02:01G	-	-	-	-	-	03:01	05:01	04:02	04:02
YG6AVBCDP	01:02	03:01	01:01:02G	-	-	-	-	-	02:01	05:01	01:01	03:01
YJ5U47CSQ	15:02	15:02	-	-	-	-	01:02:01G	-	06:01	06:01	02:01	26:01
Yj7AFgWq1	04:04	14:01	02:01:01G	-	01:01:01G	-	-	-	03:02	05:03	04:01	15:01
YKSZG8364	08:01	15:01	-	-	-	-	01:01:01G	-	04:02	06:02	04:01	04:01
YM6AC24SN	11:01	11:04	02:02:01G	-	-	-	-	-	03:01	03:01	04:01	04:02
YMZ4JQ865	07:01	11:01	02:02:01G	-	01:01:01G	-	-	-	02:01	03:01	04:01	11:01
YQ2NABJKS	04:01	13:02	03:01:01G	-	01:01:01G	-	-	-	03:02	06:04	04:01	04:01
YQR5E4P39	01:01	14:01	02:02:01G	-	-	-	-	-	05:01	05:03	02:01	04:01
YQTF2VARD	01:01	11:02	02:02:01G	-	-	-	-	-	03:01	05:01	04:01	11:01
yRe7W4pUG	13:02	15:01	03:01:01G	-	-	-	01:01:01G	-	06:02	06:04	04:01	04:01
YrnUjMTQ4	09:01	12:01	01:01:02G	-	01:01:01G	-	-	-	03:01	03:03	02:02	05:01
YS857MXT3	08:01	11:01	02:02:01G	-	-	-	-	-	03:01	04:02	04:01	15:01
YSDTN8GKE	04:01	07:01	-	-	01:01:01G	-	-	-	03:02	03:03	02:01	04:01
ySfxdRv9G	01:01	11:01	02:02:01G	-	-	-	-	-	03:01	05:01	02:01	04:01
YSJE4W3UR	01:01	01:01	-	-	-	-	-	-	05:01	05:01	04:01	04:02
YSMQCBGAU	15:01	15:02	-	-	-	-	01:01:01G	01:02:01G	06:01	06:02	04:01	04:01
YSTNBP9XV	13:01	15:02	02:02:01G	-	-	-	01:01:01G	-	05:02	06:03	01:01	04:01
YU29D6Q3V	04:04	11:01	02:02:01G	-	01:01:01G	-	-	-	03:01	03:02	04:01	04:01
YV9FXRPG8	01:01	08:01	-	-	-	-	-	-	04:02	05:01	03:01	04:01
YVN7XMBSD	07:01	16:02	-	-	01:01:01G	-	02:02:01G	-	02:01	03:01	02:01	04:02
YWB5EMQ3C	04:01	11:01	02:02:01G	-	01:01:01G	-	-	-	03:01	03:01	04:01	11:01
YWMDVQANK	03:01	07:01	02:02:01G	-	01:01:01G	-	-	-	02:01	02:01	02:02	11:01
YZDUW9V82	07:01	13:04	02:02:01G	-	01:01:01G	-	-	-	02:01	03:01	02:01	17:01
Z37MRHUX5	13:02	15:02	03:01:01G	-	-	-	01:02:01G	-	06:01	06:04	02:01	04:01
Z3N9PEB57	04:04	11:01	02:02:01G	-	01:03:03	-	-	-	03:01	03:02	04:01	04:01

Z752MXJKY	01:01	15:01	-	-	-	-	01:01:01G	-	05:01	06:02	04:01	04:01
Z794FR63N	04:03	08:03	-	-	01:01:01G	-	-	-	03:02	03:11	02:01	26:01
Z7DCPRWX5	13:01	14:06	01:01:02G	-	-	-	-	-	03:01	06:03	02:01	04:02
Z8FGW9J46	07:01	13:03	01:01:02G	-	01:01:01G	-	-	-	03:01	03:03	03:01	13:01
Z8U5RD9CV	08:09	13:01	01:01:02G	-	-	-	-	-	04:02	06:03	02:01	05:01
Z9CXG5TQ4	04:01	04:04	-	-	01:01:01G	-	-	-	03:01	03:02	03:01	04:01
ZB6WP5NHX	03:01	04:04	01:01:02G	-	01:01:01G	-	-	-	02:01	03:02	01:01	04:01
ZDEUQN5BS	03:02	07:01	02:02:01G	-	01:01:01G	-	-	-	02:01	04:02	01:01	11:01
ZDFYKAPNH	04:01	13:02	03:01:01G	-	01:01:01G	-	-	-	03:01	06:04	02:01	04:02
Zdm3BKVqM	03:01	11:01	01:01:02G	02:02:01G	-	-	-	-	02:01	03:01	02:01	13:01
ZFmJUkSXn	04:05	13:02	03:01:01G	-	01:01:01G	-	-	-	03:02	06:09	04:01	17:01
ZHRQES2X4	03:01	14:01	02:01:01G	02:02:01G	-	-	-	-	02:01	05:03	03:01	04:01
ZJEPX4HS5	11:02	13:02	02:02:01G	03:01:01G	-	-	-	-	03:01	06:04	04:01	29:01
ZJESHMV8Q	01:01	07:01	-	-	01:01:01G	-	-	-	03:03	05:01	04:01	04:02
ZKYFGWCMU	03:01	11:01	02:02:01G	-	-	-	-	-	02:01	03:01	02:02	04:02
ZP3MB96WK	03:01	04:02	02:02:01G	-	01:01:01G	-	-	-	02:01	03:02	04:01	04:02
ZQ7J2VE36	01:01	14:01	02:02:01G	-	-	-	-	-	05:01	05:03	01:01	04:01
ZRAWS6CEY	07:01	11:01	02:02:01G	-	01:01:01G	-	-	-	03:01	03:03	02:01	13:01
ZT3276DXE	04:11	15:03	-	-	01:01:01G	-	01:01:01G	-	03:02	06:02	04:02	04:02
ZtxSYmgyT	01:01	03:01	01:01:02G	-	-	-	-	-	02:01	05:01	04:01	04:01
ZUFWYK3RM	08:02	11:04	02:02:01G	-	-	-	-	-	03:01	04:02	04:02	04:02
ZURJY5MCS	01:02	04:03	-	-	01:01:01G	-	-	-	03:02	05:01	04:01	04:02
ZV2M8S576	07:01	11:01	02:02:01G	-	01:01:01G	-	-	-	03:01	03:03	04:01	04:02
ZX2PSMBHU	07:01	09:01	-	-	01:01:01G	-	-	-	02:01	03:03	03:01	04:02
Zx3CcTpzg	03:01	07:01	01:01:02G	-	01:01:01G	-	-	-	02:01	02:01	03:01	04:01
ZX6PRFWV5	07:01	11:04	02:02:01G	-	01:01:01G	-	-	-	02:01	03:01	03:01	04:01
ZX8E7NB9D	11:04	15:01	02:02:01G	-	-	-	01:01:01G	-	03:01	06:02	04:02	15:01
ZY43N8AHK	13:01	15:01	01:01:02G	-	-	-	01:01:01G	-	06:02	06:03	04:01	04:01
ZYBF7QM9U	13:01	13:01	02:02:01G	-	-	-	-	-	06:03	06:03	02:01	04:01
ZYP7BSAQ5	03:02	13:01	01:01:02G	-	-	-	-	-	04:02	06:03	01:01	04:01

Table S3: Univariate Results for non-HLA Variables in all Severe HA Participants

	Proportion Inhibitor Ever (%)	Univariate p-value	Included in Multivariate Model
**Race/Ethnicity**			
Asian	12/34 (35.29)	0.984	
Black or African American/Not Hispanic	35/83 (42.17)	0.170	Yes
Hispanic	39/79 (49.37)	0.006	Yes
Mixed Race	2/7 (28.57)	0.703	
Native Hawaiian or Other Pacific Islander	1/3 (33.33)	0.939	
None	1/3 (33.33)	0.939	
**Pathenogenic Variant Type**			
Frameshift	30/101 (29.7)	0.187	Yes
I1_inversion	7/15 (46.67)	0.362	
Large structural change (>50 bp)	21/33 (63.64)	0.001	Yes
Missense	15/108 (13.89)	0.000	Yes
Nonsense	32/74 (43.24)	0.137	Yes
Small structural change (in-frame, <50 bp)	1/5 (20)	0.479	
Splice Site change	7/18 (38.89)	0.758	

Table S4: Univariate Results for HLA Variables in all Severe HA Participants

	Proportion Inhibitor Ever (%)	Univariate p-value	Included in Multivariate Model
**HLA-DRB1**			
DRB1_0101	27/90 (30)	0.242	Yes
DRB1_0102	7/18 (38.89)	0.758	
DRB1_0103	2/13 (15.38)	0.146	Yes
DRB1_0301	33/93 (35.48)	0.995	
DRB1_0302	5/19 (26.32)	0.401	
DRB1_0401	23/70 (32.86)	0.629	
DRB1_0402	4/14 (28.57)	0.587	
DRB1_0403	4/13 (30.77)	0.721	
DRB1_0404	18/40 (45)	0.195	Yes
DRB1_0405	8/14 (57.14)	0.096	Yes
DRB1_0407	5/26 (19.23)	0.086	Yes
DRB1_0410	1/1 (100)	0.979	
DRB1_0411	2/3 (66.67)	0.290	
DRB1_0701	51/145 (35.17)	0.935	
DRB1_0801	3/15 (20)	0.217	Yes
DRB1_0802	4/11 (36.36)	0.949	
DRB1_0803	2/6 (33.33)	0.913	
DRB1_0804	5/14 (35.71)	0.984	
DRB1_0901	9/26 (34.62)	0.927	
DRB1_1001	6/15 (40)	0.710	
DRB1_1101	31/75 (41.33)	0.257	
DRB1_1102	2/7 (28.57)	0.703	
DRB1_1103	3/5 (60)	0.269	
DRB1_1104	3/28 (10.71)	0.011	Yes
DRB1_1110	1/1 (100)	0.979	
DRB1_1201	12/29 (41.38)	0.496	
DRB1_1202	5/10 (50)	0.340	
DRB1_1301	22/66 (33.33)	0.703	
DRB1_1302	22/71 (30.99)	0.403	
DRB1_1303	7/15 (46.67)	0.362	
DRB1_1304	1/5 (20)	0.479	
DRB1_1305	2/3 (66.67)	0.290	
DRB1_1331	1/1 (100)	0.979	
DRB1_1401	5/19 (26.32)	0.401	
DRB1_1402	1/4 (25)	0.664	
DRB1_1404	1/4 (25)	0.664	
DRB1_1406	1/2 (50)	0.671	
DRB1_1501	55/122 (45.08)	0.014	Yes
DRB1_1502	7/19 (36.84)	0.898	
DRB1_1503	13/21 (61.9)	0.014	Yes
DRB1_1601	4/11 (36.36)	0.949	
DRB1_1602	4/9 (44.44)	0.572	
**HLA-DRB3**			
DRB3_0101	43/144 (29.86)	0.109	Yes
DRB3_0202	77/214 (35.98)	0.843	
DRB3_0301	29/83 (34.94)	0.916	
**HLA-DRB4**			
DRB4_0101	108/311 (34.73)	0.701	
DRB4_0103	1/2 (50)	0.671	
DRB4_0107	1/1 (100)	0.979	
**HLA-DRB5**			
DRB5_0101	68/146 (46.58)	0.001	Yes
DRB5_0102	5/13 (38.46)	0.819	

DRB5_0103	1/1 (100)	0.979	
DRB5_0202	7/19 (36.84)	0.898	
**HLA-DQB1**			
DQB1_0201	73/203 (35.96)	0.855	
DQB1_0301	66/205 (32.2)	0.232	Yes
DQB1_0302	44/131 (33.59)	0.614	
DQB1_0303	21/61 (34.43)	0.859	
DQB1_0304	2/2 (100)	0.981	
DQB1_0402	14/50 (28)	0.252	
DQB1_0501	48/150 (32)	0.309	
DQB1_0502	14/30 (46.67)	0.192	Yes
DQB1_0503	5/23 (21.74)	0.169	Yes
DQB1_0504	1/3 (33.33)	0.939	
DQB1_0601	6/18 (33.33)	0.848	
DQB1_0602	75/150 (50)	0.000	Yes
DQB1_0603	19/58 (32.76)	0.652	
DQB1_0604	13/43 (30.23)	0.459	
DQB1_0608	1/1 (100)	0.979	
DQB1_0609	7/22 (31.82)	0.717	
**HLA-DPB1**			
DPB1_0101	30/91 (32.97)	0.591	
DPB1_0201	44/134 (32.84)	0.473	
DPB1_0202	8/10 (80)	0.011	Yes
DPB1_0301	36/116 (31.03)	0.269	
DPB1_0401	117/311 (37.62)	0.256	
DPB1_0402	52/147 (35.37)	0.981	
DPB1_0501	16/44 (36.36)	0.896	
DPB1_0601	5/15 (33.33)	0.862	
DPB1_0901	2/8 (25)	0.538	
DPB1_1001	10/25 (40)	0.628	
DPB1_1101	9/34 (26.47)	0.263	
DPB1_1301	11/33 (33.33)	0.793	
DPB1_1401	6/19 (31.58)	0.720	
DPB1_1501	3/11 (27.27)	0.569	
DPB1_1701	13/42 (30.95)	0.528	
DPB1_1801	10/20 (50)	0.173	Yes
DPB1_1901	6/8 (75)	0.036	Yes
DPB1_2001	1/2 (50)	0.671	
DPB1_2101	1/3 (33.33)	0.939	
DPB1_2301	3/9 (33.33)	0.893	
DPB1_3101	1/1 (100)	0.979	
DPB1_3901	1/2 (50)	0.671	
DPB1_8501	1/4 (25)	0.664	

Table S5: Univariate Results for non-HLA Variables in all HLA-typed Participants

	Proportion Inhibitor Ever (%)	Univariate p-value	Included in Multivariate Model
**Race/Ethnicity**			
American Indian or Alaska Native	0/7 (0)	0.979	
Asian	14/43 (32.56)	0.264	
Black or African American/Not Hispanic	40/106 (37.74)	0.002	Yes
Hispanic	39/141 (27.66)	0.482	
Mixed Race	2/9 (22.22)	0.832	
Native Hawaiian or Other Pacific Islander	1/3 (33.33)	0.749	
None	1/6 (16.67)	0.630	
**Disease Severity**			
Moderate	17/136 (12.5)	0.000	Yes
Severe	217/612 (35.46)	0.000	Yes
**Pathenogenic Variant Type**			
5'Upstream	0/2 (0)	0.974	
Frameshift	31/106 (29.25)	0.321	
I1_inversion	7/16 (43.75)	0.096	Yes
Large structural change (>50 bp)	22/35 (62.86)	0.000	Yes
Missense	45/439 (10.25)	0.000	Yes
None_Reported	0/3 (0)	0.979	
Nonsense	32/76 (42.11)	0.001	Yes
Small structural change (in-frame, <50 bp)	1/5 (20)	0.786	
Splice Site change	7/23 (30.43)	0.566	
Synonymous	0/25 (0)	0.974	

Table S6: Univariate Results for HLA Variables in all HLA-typed Participants

	Proportion Inhibitor Ever (%)	Univariate p-value	Included in Multivariate Model
**HLA-DRB1**			
DRB1_0101	31/150 (20.67)	0.160	Yes
DRB1_0102	9/32 (28.12)	0.706	
DRB1_0103	2/20 (10)	0.131	Yes
DRB1_0301	36/158 (22.78)	0.433	
DRB1_0302	5/23 (21.74)	0.693	
DRB1_0304	0/2 (0)	0.974	
DRB1_0401	28/122 (22.95)	0.528	
DRB1_0402	4/17 (23.53)	0.867	
DRB1_0403	4/22 (18.18)	0.442	
DRB1_0404	24/68 (35.29)	0.051	Yes
DRB1_0405	9/19 (47.37)	0.031	Yes
DRB1_0407	5/45 (11.11)	0.032	Yes
DRB1_0408	0/5 (0)	0.973	
DRB1_0410	1/2 (50)	0.443	
DRB1_0411	3/9 (33.33)	0.579	
DRB1_0438	0/1 (0)	0.981	
DRB1_0456	0/1 (0)	0.981	
DRB1_0701	55/211 (26.07)	0.766	
DRB1_0801	5/31 (16.13)	0.240	Yes
DRB1_0802	5/22 (22.73)	0.781	
DRB1_0803	3/9 (33.33)	0.579	
DRB1_0804	5/19 (26.32)	0.916	
DRB1_0806	0/3 (0)	0.979	
DRB1_0809	0/1 (0)	0.981	
DRB1_0901	10/37 (27.03)	0.803	
DRB1_1001	6/24 (25)	0.975	
DRB1_1101	38/122 (31.15)	0.112	Yes
DRB1_1102	2/11 (18.18)	0.589	
DRB1_1103	4/10 (40)	0.291	
DRB1_1104	5/56 (8.93)	0.007	Yes
DRB1_1110	1/1 (100)	0.978	
DRB1_1111	0/1 (0)	0.981	
DRB1_1201	14/41 (34.15)	0.185	Yes
DRB1_1202	5/11 (45.45)	0.134	Yes
DRB1_1301	25/108 (23.15)	0.590	
DRB1_1302	29/117 (24.79)	0.897	
DRB1_1303	8/31 (25.81)	0.945	
DRB1_1304	1/7 (14.29)	0.511	
DRB1_1305	2/4 (50)	0.277	
DRB1_1312	0/1 (0)	0.981	
DRB1_1331	1/1 (100)	0.978	
DRB1_1401	8/36 (22.22)	0.668	
DRB1_1402	1/6 (16.67)	0.630	
DRB1_1404	1/7 (14.29)	0.511	
DRB1_1405	0/2 (0)	0.974	
DRB1_1406	1/6 (16.67)	0.630	
DRB1_1501	61/204 (29.9)	0.089	Yes
DRB1_1502	7/26 (26.92)	0.845	
DRB1_1503	16/30 (53.33)	0.001	Yes
DRB1_1601	6/17 (35.29)	0.342	
DRB1_1602	4/12 (33.33)	0.521	
**HLA-DRB3**			
DRB3_0101	48/240 (20)	0.032	Yes

DRB3_0201	0/6 (0)	0.970	
DRB3_0202	90/349 (25.79)	0.785	
DRB3_0210	1/1 (100)	0.978	
DRB3_0301	36/130 (27.69)	0.497	
**HLA-DRB4**			
DRB4_0101	124/493 (25.15)	0.929	
DRB4_0103	1/2 (50)	0.443	
DRB4_0107	1/1 (100)	0.978	
**HLA-DRB5**			
DRB5_0101	76/237 (32.07)	0.006	Yes
DRB5_0102	5/19 (26.32)	0.916	
DRB5_0103	1/1 (100)	0.978	
DRB5_0202	9/28 (32.14)	0.399	
**HLA-DQB1**			
DQB1_0201	79/316 (25)	0.891	
DQB1_0301	75/345 (21.74)	0.062	Yes
DQB1_0302	56/215 (26.05)	0.769	
DQB1_0303	23/92 (25)	0.949	
DQB1_0304	2/2 (100)	0.969	
DQB1_0305	0/1 (0)	0.981	
DQB1_0311	0/1 (0)	0.981	
DQB1_0401	0/2 (0)	0.974	
DQB1_0402	17/80 (21.25)	0.389	
DQB1_0501	54/242 (22.31)	0.224	Yes
DQB1_0502	16/38 (42.11)	0.017	Yes
DQB1_0503	9/45 (20)	0.406	
DQB1_0504	1/3 (33.33)	0.749	
DQB1_0601	7/27 (25.93)	0.937	
DQB1_0602	83/241 (34.44)	0.000	Yes
DQB1_0603	22/100 (22)	0.427	
DQB1_0604	20/80 (25)	0.953	
DQB1_0608	1/1 (100)	0.978	
DQB1_0609	7/30 (23.33)	0.804	
DQB1_0611	0/2 (0)	0.974	
DQB1_0633	0/1 (0)	0.981	
**HLA-DPB1**			
DPB1_0101	31/131 (23.66)	0.649	
DPB1_0201	53/216 (24.54)	0.778	
DPB1_0202	9/18 (50)	0.020	Yes
DPB1_0301	40/185 (21.62)	0.206	Yes
DPB1_0401	135/554 (24.37)	0.461	
DPB1_0402	67/255 (26.27)	0.671	
DPB1_0501	20/62 (32.26)	0.194	Yes
DPB1_0601	8/32 (25)	0.971	
DPB1_0901	3/11 (27.27)	0.878	
DPB1_1001	11/30 (36.67)	0.150	Yes
DPB1_1101	9/53 (16.98)	0.158	Yes
DPB1_1301	13/47 (27.66)	0.700	
DPB1_1401	6/30 (20)	0.501	
DPB1_1501	3/13 (23.08)	0.854	
DPB1_1601	0/9 (0)	0.976	
DPB1_1701	14/54 (25.93)	0.910	
DPB1_1801	12/26 (46.15)	0.016	Yes
DPB1_1901	7/14 (50)	0.041	Yes
DPB1_2001	1/4 (25)	0.990	
DPB1_2101	1/3 (33.33)	0.749	
DPB1_2301	3/10 (30)	0.730	

DPB1_2601	0/4 (0)	0.976
DPB1_2701	0/2 (0)	0.974
DPB1_2801	0/1 (0)	0.981
DPB1_2901	0/3 (0)	0.979
DPB1_3101	1/1 (100)	0.978
DPB1_3550	0/1 (0)	0.981
DPB1_3801	0/1 (0)	0.981
DPB1_3901	1/2 (50)	0.443
DPB1_6301	0/1 (0)	0.981
DPB1_8501	1/6 (16.67)	0.630
DPB1_9150	0/1 (0)	0.981
